# Safety and tolerability of the protein C activator AB002 in end-stage renal disease patients on hemodialysis: a randomized phase 2 trial

**DOI:** 10.1038/s43856-024-00575-y

**Published:** 2024-07-26

**Authors:** Norah G. Verbout, Christina U. Lorentz, Brandon D. Markway, Michael Wallisch, Thomas C. Marbury, Enrico Di Cera, Joseph J. Shatzel, András Gruber, Erik I. Tucker

**Affiliations:** 1 Aronora, Inc., Portland, OR USA; 2 Department of Biomedical Engineering, Oregon Health & Science University, Portland, OR USA; 3 Orlando Clinical Research Center, Orlando, FL USA; 4 Edward A. Doisy Department of Biochemistry and Molecular Biology, School of Medicine, Saint Louis University, St. Louis, MO USA

**Keywords:** Haemodialysis, Recombinant protein therapy, Drug development

## Abstract

**Background:**

The protein C system regulates blood coagulation, inflammation, and vascular integrity. AB002 is an injectable protein C activating enzyme under investigation to safely prevent and treat thrombosis. In preclinical models, AB002 is antithrombotic, cytoprotective, and anti-inflammatory. Since prophylactic use of heparin is contraindicated during hemodialysis in some end-stage renal disease (ESRD) patients, we propose using AB002 as a short-acting alternative to safely limit blood loss due to clotting in the dialysis circuit.

**Methods:**

This phase 2, randomized, double-blind, placebo-controlled, single-dose study evaluates the safety and tolerability of AB002 administered into the hemodialysis line of ESRD patients during hemodialysis at one study center in the United States (ClinicalTrials.gov: NCT03963895). In this study, 36 patients were sequentially enrolled into two cohorts and randomized to AB002 or placebo in a 2:1 ratio. In cohort 1, patients received 1.5 µg/kg AB002 (*n* = 12) or placebo (*n* = 6); in cohort 2, patients received 3 µg/kg AB002 (*n* = 12) or placebo (*n* = 6). Patients underwent five heparin-free hemodialysis sessions over 10 days and were dosed with AB002 or placebo during session four.

**Results:**

Here we show that AB002 is safe and well-tolerated in ESRD patients, with no treatment-related adverse events. Clinically relevant bleeding did not occur in any patient, and the time to hemostasis at the vascular access sites is not affected by AB002.

**Conclusions:**

As far as we are aware, this proof-of-concept study is the first clinical trial assessing the therapeutic potential of protein C activation. The results herein support additional investigation of AB002 to safely prevent and treat thrombosis in at-risk populations.

## Plain language summary

Some people with kidney disease require hemodialysis, a process in which a machine filters the blood to remove waste products. The process of hemodialysis can trigger blood clotting in the hemodialysis circuit. Therefore, the blood-thinner heparin is commonly used to prevent blood from clotting. However, some patients cannot tolerate heparin. Here we describe a clinical trial in which we tested whether a drug called AB002 is safe and can reduce hemodialysis circuit clotting in people with permanent kidney disease (end-stage renal disease) undergoing hemodialysis. AB002 appears to be safe and well-tolerated, and we observed reduced clotting without any signs of increased bleeding. Further studies are required in more patients to determine whether AB002 can be used routinely during hemodialysis to safely prevent or treat blood clots.

## Abstract (web summary)

Verbout et al. report findings from a phase 2 proof-of-concept study of the protein C activator AB002 in patients with end stage renal disease undergoing heparin-free hemodialysis. AB002 appears safe and well-tolerated, while demonstrating reduced dialyzer clot severity and no increased bleeding at the vascular access site compared to placebo.

## Introduction

AB002 (E-WE thrombin variant (recombinant)) is an engineered thrombin analog that selectively activates the natural antithrombotic and cytoprotective enzyme, protein C, on the endothelial surface and on pathological blood clots^1–4^. Activated protein C (APC) anticoagulates blood by inhibiting thrombin generation through enzymatic degradation of coagulation factors Va and VIIIa. In primates, AB002 reduces thrombogenesis by locally downregulating thrombin generation without causing systemic anticoagulation that can impair hemostasis^5–8^. In a phase 1 trial of healthy volunteers, AB002 was found to have a favorable safety profile, with no serious adverse events at a dose-range of 0.5–4 µg/kg^8^. Since AB002 is not renally metabolized, has both antithrombotic and anti-inflammatory activity, and is not expected to increase bleeding, it could represent a substantial advance for safer short-term thromboprophylaxis in end-stage renal disease (ESRD) and other patients at high bleeding risk, including those who cannot tolerate heparin anticoagulation.

The global prevalence of chronic kidney disease (CKD) has been estimated to be 13.4%, according to a recent systematic review^9^. CKD has a high risk for progression to ESRD, a condition requiring renal replacement therapies, such as dialysis or kidney transplantation, to maintain survival. In patients with ESRD requiring hemodialysis, exposure of blood to foreign surfaces within the hemodialysis circuit activates coagulation and causes blood loss due to entrapment. Anemia is a common complication of ESRD, with approximately 85% of hemodialysis patients requiring erythropoiesis-stimulating agents^10^. Despite this, many ESRD patients have erythropoietin-resistant anemia and/or anemia of inflammation and cannot tolerate additional blood loss during hemodialysis, which would further worsen their anemia, prognosis, and quality of life^11–13^.

The most commonly employed anticoagulant for reducing hemodialysis circuit clotting is unfractionated heparin. Heparin reduces patient blood loss due to blood entrapment in the hemodialysis circuit, as well as the incidence of circuit changeouts resulting from complete dialyzer occlusion^14^. However, approximately 10% of ESRD patients do not receive heparin during hemodialysis due to heparin-resistance, heparin intolerance, increased bleeding risk, or cultural preferences^15^. In patients undergoing heparin-free hemodialysis, the incidence of dialyzer clotting has been reported to occur in up to 10% of dialysis sessions^16^. When a circuit changeout occurs, it is estimated that up to 180 mL blood is lost, attributable to the fill volume of the dialyzer and tubing^16^. While saline flushes can be used to maintain circuit patency during heparin-free hemodialysis, they can reduce dialyzer efficiency and increase patient fluid volume^17,18^.

To address this need for a safe, heparin-free strategy to prevent hemodialysis circuit clotting in this patient population, we conducted a phase 2 proof-of-concept clinical trial to evaluate the safety and preliminary antithrombotic efficacy of AB002 administration (NCT03963895). Here, we show that AB002 demonstrates a favorable safety profile, with no treatment-related adverse events (AE) and no clinically relevant bleeding. Additionally, in patients receiving AB002, clotting severity in the dialysis circuit is significantly reduced, the frequency of thrombo-occlusive events requiring changeout is numerically reduced, and exploratory biochemical markers indicative of clot generation are reduced.

## Methods

### Study design

This was a phase 2, randomized, double-blind, placebo-controlled, single-dose study of AB002 designed to evaluate the safety and efficacy of two dose levels administered into the proximal hemodialysis line to ESRD patients during maintenance hemodialysis. The study was conducted in compliance with ICH Good Clinical Practice guidelines and was performed at the Orlando Clinical Research Center (Orlando, FL). The first patient was enrolled on July 3, 2019, and the last patient was enrolled on November 13, 2020. The clinical trial protocol (Supplementary Methods) and all amendments were approved by IntegReview IRB (Institutional Review Board) (now operating under Advarra) prior to study start and informed consent was obtained from all participants prior to study-specific procedures. The study is registered at ClinicalTrials.gov as NCT03963895.

### Study population

Study participants were recruited from the Orlando, FL region using a site database and media advertisements. ESRD patients between 18 and 80 years old were eligible for enrollment if they were on a stable outpatient hemodialysis regimen ( > 3 months) at least 3 times per week using an arteriovenous (AV) fistula, AV graft or central venous catheter. A full list of inclusion and exclusion criteria is provided below.

### Inclusion and exclusion criteria

Patients fulfilling the following criteria were included: (1) ESRD maintained on stable outpatient HD regimen, using an established ( > 3 months) and normally functioning, regular flow, uninfected mature AV fistula (or AV graft) and skin consistent with standard chronic HD access injuries, and HD stability defined as Kt/V ≥ 1.2 within 3 months prior to screening at a healthcare center for > 3 months from screening; (2) on HD regimen at least 3 times per week for a minimum of 3 hours per dialysis session, using a complication-free well maintained AV fistula (or AV graft), expected and plan to continue this throughout and for at least 3 months beyond the study; (3) capable of understanding the written informed consent, provides signed and witnessed written informed consent and agrees to comply with protocol requirements and study-related procedures; (4) willing to be confined to the CRU for the duration of the study, able to comply with all study-related requirements, and able to adhere to study restrictions and visit schedules; (5) male or female, between 18 and 80 years of age (inclusive) at the time of screening; (6) BMI of ≥ 18 at the time of screening; (7) considered by the PI to be clinically stable with respect to underlying ESRD, based on the medical evaluation that includes medical and surgical history, and a complete physical examination including vital sign measurements, ECGs, and clinical laboratory and coagulation test results at screening. Repeat assessments are permitted for any laboratory, coagulation, ECG, or vital sign parameter required for enrollment; (8) female patients must be of non-childbearing potential and must have undergone one of the following sterilization procedures at least 6 months prior to dosing: hysteroscopic sterilization, bilateral tubal ligation or bilateral salpingectomy, hysterectomy, bilateral oophorectomy, or be postmenopausal with amenorrhea for at least 1 year prior to dosing and follicle-stimulating hormone (FSH) serum levels consistent with postmenopausal status as per PI or designee judgment; and (9) male patients must either be sterile (vasectomy with history of a negative sperm count following the procedure); practice total abstinence from sexual intercourse as the preferred lifestyle (periodic abstinence is not acceptable); use a male condom with any sexual activity; or agree to use a birth control method considered to be appropriate by the Investigator from the time of dosing until 90 days after study drug administration. Male patients must agree not to donate sperm for a period of 90 days after study drug administration.

Patients fulfilling any of the following criteria were excluded: (1) documented history of acute vasoocclusive thrombotic event (acute coronary syndrome, stroke or transient ischemic attack, venous thromboembolic event), or vascular access fistula or AV graft failure in the past 3 months; (2) with the exception of unfractionated heparin during HD that is allowed until study check-in, concomitant or prior use of anticoagulant/antiplatelet agents (e.g., low molecular weight heparins, warfarin, apixaban, bivalirudin, ticagrelor, edoxaban, dabigatran, rivaroxaban, clopidogrel, prasugrel, ticlopidine, eptifibatide, tirofiban, dipyridamole, diclofenac, and all other non steroidal anti-inflammatory drugs) that may affect hemostasis for 2 weeks prior to check-in on day −8 and throughout the study; (3) any clinically significant (CS) concomitant disease or condition (including treatment for such conditions) that, in the opinion of the PI, could either interfere with the study drug, compromise interpretation of study data, or pose an unacceptable risk to the patient; (4) any other CS abnormalities in laboratory test results at screening or day −8 check-in that would, in the opinion of the PI, increase the patient’s risk of participation, jeopardize complete participation in the study, or compromise interpretation of study data; (5) pregnant (positive pregnancy test) at screening or check-in on day −8. If serum human chorionic gonadotropin (hCG) pregnancy test results are indeterminate, follow-up testing should be performed to determine eligibility. All female patients will not be pregnant and will have a negative pregnancy test at screening and check-in on day −8, with the following exception: females receiving dialysis with an indeterminate pregnancy test result or persistently low hCG resulting in a false positive pregnancy test may be included in the study at the discretion of the PI. Postmenopausal patients with a result outside the postmenopausal range or an indeterminate pregnancy test will undergo additional testing with FSH to confirm postmenopausal status prior to study enrollment; (6) treatment with another investigational drug or participation in a device study within 30 days (or 5 half-lives, whichever is longer) prior to check-in on day −8; (7) acute illness that is considered by the PI to be CS within 2 weeks of check-in on day −8; (8) surgery within the past 90 days prior to dosing which in the opinion of the PI or designee is clinically relevant; (9) currently have established underlying inherited or acquired symptomatic bleeding disorders and/or are at risk for excessive bleeding per PI judgment or current active bleeding (e.g., gastrointestinal, intracranial), aside from minor bleeding from the puncture site on the AV fistula or AV graft, which would be expected to occur during the dialysis procedure, with the following values: platelet count < 100,000 cells/mm^3^ (if < 100,000 cells/mm^3^ but > 75,000 cells/mm^3^, with permission of PI and medical monitor) at screening or check-in on day −8, INR > 1.4 at screening or check-in on day −8, aPTT up to 1.2 x upper limit of normal (ULN) (if > 1.2 x ULN and up to < 1.5 x ULN, with permission of PI and medical monitor) at screening or check-in on day −8, alanine aminotransferase (ALT) or aspartate aminotransferase (AST) > 2 x ULN at screening or check-in on day −8, total bilirubin > 1.2 x ULN at screening or check-in on day −8; (10) seated blood pressure < 90/40 mmHg at screening and check-in on day −8; (11) exclusion criteria for ECG at screening and check-in on day −8: heart rate < 45 and > 110 bpm, QTcF interval > 500 msec, any significant arrhythmia or conduction abnormality, (including but not specific to atrioventricular block [2^nd^ degree or higher], Wolff Parkinson White syndrome [unless curative radio ablation therapy]), which, in the opinion of the PI and Medical Monitor, could interfere with the safety for the individual patient, non-sustained or sustained ventricular tachycardia ( > 2 consecutive ventricular ectopic beats at a rate of > 1.7/second); (12) history of a CS allergy or a known sensitivity or idiosyncratic reaction to any compound known to be present in E-WE thrombin, its related compounds, or any compound listed as being present in the study formulation; (13) hypersensitivity to ß-lactam / penicillin derivatives; (14) participate in strenuous exercise from 72 h prior to check-in on day −8 and throughout the study; (15) positive test for drugs of abuse and/or positive alcohol test at screening or check-in on day −8 if not accounted for by a prescription medication. Patients with a positive test based on a prescribed medication may be enrolled; (16) positive test at screening for hepatitis B surface antigen (HBsAg) or human immunodeficiency virus (HIV). If a patient with ESRD has positive test results for hepatitis C virus (HCV) but liver function tests are otherwise not clinically significant, the patient may be included at the PI’s discretion; (17) receiving blood purification therapy other than HD; (18) donation of blood or significant blood loss within 56 days prior to dosing; (19) plasma donation within 7 days prior to dosing; (20) presence of advanced malignant neoplasms of any organ or system that produces illness or symptoms that have been treated within 3 months with chemotherapy or whole body irradiation, or bone marrow irradiation, and may affect life expectancy in the following 6 months; and (21) any other reason that would render the patient unsuitable for study enrollment at the discretion of the PI.

After trial commencement, the study protocol was amended to remove an exclusion criterion that specified a lower limit value for hemoglobin concentration measured at screening and check-in. The IRB reviewed this amendment and determined that removal of this criterion would not affect patients’ safety upon drug administration and would not interfere with study endpoints. No interim analysis was performed. Patient participation in the study was subject to stopping guidelines provided in the study protocol (Supplementary Methods).

### Randomization and dosing

A Celerion statistician generated a random allocation sequence for each cohort at a 2:1 allocation ratio of drug and placebo using a computerized randomization scheme. In each cohort, patients were assigned a unique randomization number at the time of dosing and received the corresponding treatment according to the randomization schedule, with male patients assigned consecutively upward starting at 1 (cohort 1) and 19 (cohort 2), and female patients assigned consecutively downward starting at 18 (cohort 1) and 36 (cohort 2). Block randomization was not utilized. The blinding of the study was maintained as follows: the randomization schedule was available only to the clinical site pharmacy staff preparing the drug who were not involved in any other aspect of the study, including drug administration. The randomization schedule was not available to the Sponsor, patients, study PI, or staff members responsible for monitoring and evaluating patients. The study drug and the matching placebo were indistinguishable in appearance.

In this parallel study, patients were randomly assigned to active drug or matching placebo in a 2:1 allocation ratio. Patients were enrolled into two separate cohorts, with cohort 1 completed before initiation of cohort 2. In cohort 1, the dose level was 1.5 µg/kg and in cohort 2, the dose level was 3 µg/kg. A single dose of AB002 or placebo was administered as a bolus (1/3 of dose) followed by infusion (2/3 of dose) into the dialysis line during a 4 h hemodialysis procedure.

**Fig. 1:**
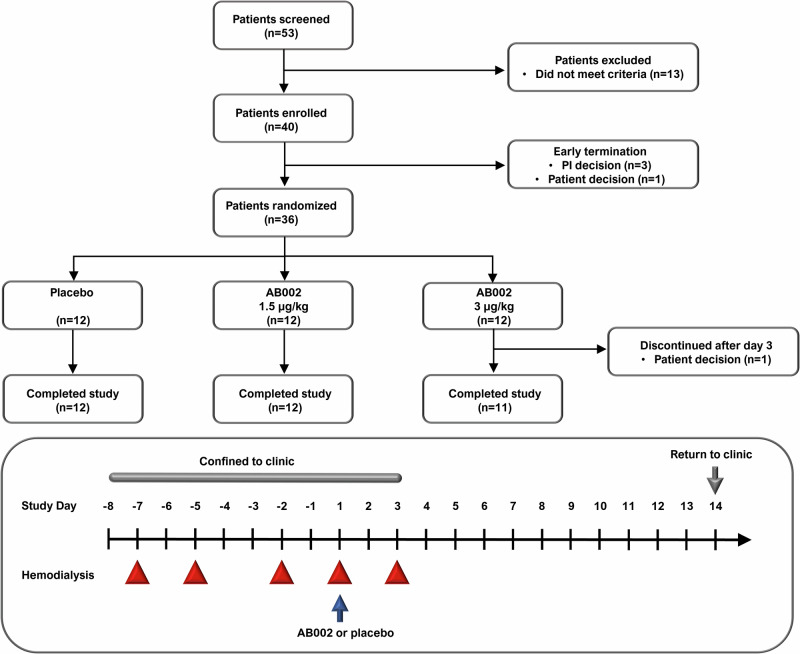
Enrollment, randomization, and study timeline. Fifty-three patients were screened for study eligibility. Thirteen patients did not meet the inclusion/exclusion criteria and were not enrolled in the study. An additional four patients terminated the study early, prior to randomization on study day 1. Thirty-six patients were randomized and confined to the clinic from study day −8 to study day 3 (a total of 10 days), after which they returned to the clinic on study day 14 for post-dose immunogenicity blood draw. Pre-dose hemodialysis occurred on study days −7, −5 and −2, dosing occurred on study day 1 and post-dosing hemodialysis occurred on study day 3 (red triangles). Cohorts were dosed sequentially, starting with cohort 1 (1.5 µg/kg or matching placebo). Once cohort 1 was completed, dosing for cohort 2 began (3 µg/kg or matching placebo). Placebo from both cohorts were pooled together for analysis. All 36 patients randomized were included in the safety and efficacy analyses. A total of 35 patients completed the study, with one patient that withdrew after hemodialysis on day 3 and did not complete the day 14 assessment.

Study screening, enrollment, evaluation and follow up was performed by a clinical site coordinator under the supervision of the study PI. Patients were checked-in to the clinical research unit on study day −8 and were confined through study day 3 (a total of 10 days), returning on day 14 for follow up procedures. On days −7, −5, −2, 1, and 3, patients underwent heparin-free dialysis using Fresenius Optiflux F180NR dialyzers. On study day 1 (the 4^th^ dialysis session), patients received either AB002 or placebo. Patients were assessed for all scheduled procedures and endpoints before, during and after each hemodialysis session. The study events timeline is presented in Fig. 1.

### Study outcomes and assessments

The primary outcome of this study was the safety and tolerability of AB002. Safety and tolerability outcome measures were evaluated following active treatment versus pre-treatment (at check-in on day −8) and versus placebo, and included the number and severity of AEs (including vascular access site reactions), physical examinations, bleeding time from the vascular access sites, vital sign measurements (body temperature, respiratory rate, blood pressure, and heart rate), 12-lead ECGs, clinical laboratory parameters, including hematology (hemoglobin, hematocrit, total and differential leukocyte count, red blood cell count, platelet count), serum chemistry (blood urea nitrogen, chloride, bicarbonate, albumin, creatinine, glucose, alkaline phosphatase, alanine aminotransferase, aspartate aminotransferase, direct bilirubin, total bilirubin, lactate dehydrogenase, and sodium), and coagulation (fibrinogen, prothrombin time/INR, prothrombin time, thrombin time, activated partial thromboplastin time). All patients were anuric, therefore urinalysis was not assessed.

To measure bleeding time from the vascular access site, the access needles were removed from the vascular access site at the end of hemodialysis and a pressure dressing was applied. After 10 min, the dressing was removed, and the access site was assessed for evidence of hemostasis. If bleeding was observed, the dressing was reapplied to the site and assessed every 5 min until hemostasis was achieved.

To monitor potential AB002-mediated alterations in coagulation parameters, thrombin time, prothrombin time, and activated partial thromboplastin time were additionally evaluated at study check-in (day −8), on each day of hemodialysis (prior to session and at end of session) and again at follow up on day 14 using standard laboratory assays.

An additional safety outcome of the study included development of antibodies to AB002 or wild-type (WT) thrombin after drug exposure, as measured by validated anti-drug antibody assays. Thus, plasma samples were collected on day 1 and day 14, relative to dosing, and evaluated at Prolytix (formerly Haemtech Biopharma Services) for immunogenic responses using custom validated bridging-type ELISAs for the detection and titer of anti-drug antibodies (ADA) or anti-human WT thrombin antibodies^8^.

There were three secondary outcomes defined in the study protocol, which included pharmacodynamics evaluation, hemodialysis efficiency, and antithrombotic activity. For pharmacodynamic analysis, the magnitude and duration of action of AB002 was evaluated by measuring a surrogate biomarker, plasma APC-protein C inhibitor (PCI), by a qualified ELISA (Celerion, Lincoln, NE). APC-PCI levels were measured in samples collected on day 1 prior to dosing and at 0.167, 0.5, 1, 2, 4, 6, and 24 h after initiation of dosing. In addition, plasma protein C levels on day of dosing were evaluated as an exploratory outcome at Aronora, Inc. by ELISA (Innovative Research, Inc.) from samples collected at 0, 4, 6, and 24 h post-dose.

Hemodialysis efficiency was evaluated by comparing blood urea nitrogen (BUN) and potassium levels before and after hemodialysis following AB002 treatment versus pre-treatment and placebo.

Efficacy of antithrombotic activity was measured by thrombus accumulation within the dialyzer circuit (evaluated by visual inspection) following active treatment with AB002. During hemodialysis, the circuit was monitored for the development of macrothrombi, and saline flushes were administered into the circuit as needed by a technician blinded to treatment. If complete occlusion of the hemodialysis circuit occurred, the circuit was replaced, and hemodialysis resumed. At the end of hemodialysis, the circuit (dialyzer filter, tubing, and venous chamber) was flushed with saline to return extracorporeal blood to the patient, and the dialyzer filter was drained of dialysate solution. Thrombus accumulation visible on the surface of the dialyzer filter membrane and in the venous chamber were assessed by visual examination by a technician blinded to treatment immediately following the hemodialysis session at the clinical study site. A visual scoring scale (Supplementary Table 1) was used to assign a numeric value representing the degree of clotting on the dialyzer filter surface or within the venous chamber. For the dialyzer filter, the score ranged between 1 and 5, with 1 being no observed clotting or few blood streaks affecting less than 5% of the fibers, and 5 being blood streaks affecting more than 75% of fibers on the dialyzer surface. For the venous chamber, the score ranged between 1 and 6, with 1 being no detectable clotting, and 6 being clot formation affecting more than 75% of the chamber space. In some cases, there were hemodialysis sessions in which a circuit changeout occurred and both dialyzer filters and venous chambers were scored and the numeric value for each component were combined. Complete occlusions were recorded but not assigned a numeric value. The effect of treatment on clotting severity was determined by comparing the change in mean visual clotting score values from baseline across the treatment groups. Since the duration of effect of AB002 is less than 24 h, baseline visual clotting scores were calculated for each group by averaging scores for all non-dosing days (days −7, −5, −2, and 3).

The exploratory outcomes of this study that were not prespecified in the study protocol included the effect of AB002 on the frequency and volume of saline flushes required to maintain circuit patency during hemodialysis, the frequency of hemodialysis circuit changeouts, thrombin generation during hemodialysis, and quantification of blood entrapment within the dialyzer filter at the end of hemodialysis. All exploratory outcomes were evaluated following active treatment versus placebo, with baseline values for outcome measures calculated from non-dosing days as described above for clotting severity.

To evaluate the effect of treatment on the frequency or the volume of saline flushes, the number and volume of saline flushes measured on non-dosing days were averaged for each subject and then the mean number and volume for non-dosing days calculated as the baseline for each treatment group. Mean number and volume of flushes for subjects in each group on the single day of dosing were compared to these values to calculate % baseline as a measure of change. Because some sessions had zero flushes which precludes individual % baseline calculations, no standard deviation is calculated.

To evaluate the effect of treatment on circuit patency, the total number of changeouts that occurred on non-dosing days were summed for each treatment group and compared to the total number of changeouts that occurred on the day of dosing for the respective treatment group, yielding a changeout frequency for each treatment group.

To evaluate changes in intravascular thrombin generation during hemodialysis, plasma thrombin-antithrombin (TAT) complex levels were measured at the start and end of hemodialysis at Aronora, Inc. by ELISA (TAT-micro, Enzygnost, Siemens), per manufacturer instructions.

The relative amount of blood entrapment within the membrane of the dialyzer filter was determined as previously described^19^ with some minor modifications. Briefly, each filter was thawed, and the filtrate collected and stored at 4 °C (day 1 filtrate). The dialyzer was then re-filled with distilled water, clamped shut, and stored horizontally at ambient temperature for one week. The dialyzer was rotated once mid-week and on day 7, the dialyzer was drained, and filtrate collected and combined with the day 1 filtrate. The total volume was brought up to 500 mL with distilled water and samples were analyzed for levels of potassium and iron using inductively coupled plasma mass spectrometry (ICP-MS) in kinetic energy discrimination mode (Elemental Analysis Core, Oregon Health & Science University)^20^. Dialyzer filters from a single subject were processed at Aronora, Inc. in unison by an investigator blinded to treatment status. Dialyzers in which the plastic cartridge encasing the fibers was breached were excluded from ion concentration analysis. In some cases, there were hemodialysis sessions in which a circuit changeout occurred and two dialyzers were analyzed and the concentration results for both dialyzer filters were combined. For potassium and iron concentration analysis, ion concentrations from the single day of dosing for each subject were compared to mean ion concentrations from the subject’s non-dosing days and expressed as % baseline for each subject. The ion concentration % baseline results for the subjects were then averaged across treatment groups and analyzed for statistical significance.

The efficacy analysis in this study was exploratory in nature and not powered. Data were collected for four non-dosing days (days −7, −5, −2, 3) and one dosing day (day 1) for all subjects whenever possible. In one case, hemodialysis was performed off-site, and it was not possible to count saline flushes/volume, measure circuit changeouts, determine a visual clotting score, or quantify entrapped blood within the dialyzer filter.

In addition, the fractional urea clearance, defined as Kt/V, and urea reduction ratio (URR) were calculated via methods described by the National Kidney Foundation clinical practice guidelines for hemodialysis^21^:

Kt/V = −Ln(R − 0.008 × t) + (4 − 3.5 × R) × UF/W in which Ln is the natural logarithm; R is the post-dialysis BUN ÷ pre-dialysis BUN; t is the dialysis session length in hours; UF is the ultrafiltration volume in liters; and W is the patient’s post-dialysis weight in kilograms.

URR = 100 × (1 – C_t_ /C_0_) in which C_t_ is the post-dialysis BUN and C_0_ is the pre-dialysis BUN.

### Statistics and reproducibility

The intention to treat (ITT) population included all patients that were enrolled and randomized. Safety assessment included all patients who were randomized and received study drug and included study events that occurred from check-in on day −8 through the end of study on day 14 (*n* = 36). Applicable continuous variables were summarized using descriptive statistics, including n, arithmetic mean, and SD. Data from patients that received placebo were pooled across both cohorts.

For the efficacy analysis, one-way ANOVA with Tukey pairwise comparison was used unless outcome variables failed the Shapiro-Wilk normality test or Brown-Forsythe test for equal variance. For these cases (iron, TAT, and dialyzer filter scores), data was analyzed by Kruskal-Wallis one-way ANOVA on Ranks with pairwise comparison by Tukey Test, except when unequal group sizes required Dunn’s Method (TAT and iron). Samples for all subjects (*n* = 12 per group) were analyzed whenever possible but groups for individual data sets were lower in some cases due to broken dialyzers, missing samples, or exclusion by outlier analysis (1.5x interquartile range method). Individual data set n are indicated in figure legends. For all ELISA-based data, individual samples were measured in duplicate. Only day 1 samples were analyzed for the biomarkers of drug exposure, APC-PCI and PC. For TAT, samples from all sessions were analyzed. For pre-dialysis measures, the mean of all sessions was calculated as baseline whereas post-dialysis, the mean of the four non-dosing day sessions was calculated separately from the single dosing day session. Statistical analysis was performed using SigmaPlot 11.2 or GraphPad Prism 5. A *p*-value of less than 0.05 was considered statistically significant for all tests.

### Reporting summary

Further information on research design is available in the Nature Portfolio Reporting Summary linked to this article.

## Results

### Patient disposition and baseline characteristics

Overall, 53 patients with ESRD were screened in this study, 40 of which were enrolled (Fig. 1). Of the 40 patients enrolled, a total of 36 patients were randomly assigned to treatment and included in analysis of the safety and efficacy endpoints. In addition, 4 patients attended study check-in but were not randomized; 3 patients were discontinued due to a medical condition or circumstance that exposed them to substantial risk and/or the patient was unable to adhere to the protocol requirements, and 1 patient withdrew. In addition, 1 patient that received the 3 µg/kg dose on day 1 elected to withdraw after completing hemodialysis on day 3 after the last planned hemodialysis session and did not attend the day 14 follow up. Overall, 35/36 patients (97%) completed the study through day 14. Patient demographics and baseline characteristics are provided in Table 1. The majority of patients identified as Black or African American, the mean (SD) age was 54.2 (9.91) years old, and 26/36 patients (72%) identified as male.

**Table 1:** Patient Characteristics

Trait	Category	Placebo Pooled (*n* = 12)	AB002 1.5 µg/kg (*n* = 12)	AB002 3 µg/kg (*n* = 12)
Sex	Female	3 (25%)	4 (33%)	3 (25%)
	Male	9 (75%)	8 (67%)	9 (75%)
Race	Black or African American	12 (100%)	11 (92%)	10 (83%)
	White	0 (0%)	1 (8%)	2 (17%)
Ethnicity	Hispanic or Latino	0 (0%)	1 (8%)	0 (0%)
	Not Hispanic or Latino	12 (100%)	11 (92%)	12 (100%)
Age, mean (SD), y		54.6 (12.30)	54.8 (8.29)	53.3 (9.56)
Weight, mean (SD), kg		80.2 (13.32)	86.5 (16.77)	92.3 (30.82)
BMI, mean (SD)		26.5 (3.62)	28.2 (5.69)	29.5 (9.33)
Tobacco use, *n* (%)		2 (17%)	1 (8%)	2 (17%)
Diagnosis ESRD, mean (SD), y		9.4 (7.89)	6.5 (4.38)	6.3 (4.73)
Primary cause of kidney failure, *n* (%)	Hypertension	5 (42%)	7 (58%)	4 (33%)
	Diabetes	3 (25%)	2 (17%)	4 (33%)
	Polycystic kidney disease	0 (0%)	1 (8%)	0 (0%)
	Unknown	1 (8%)	0 (0%)	0 (0%)
	Multiple	3 (25%)	2 (17%)	4 (33%)
aPTT^a^, mean (SD), s		34.4 (4.33)	35.0 (4.06)	34.7 (3.63)
Platelet count^a^, mean (SD), K/µL		205.6^b^ (76.39)	175.1 (63.57)	211.0 (57.35)
Hemoglobin^a^, mean (SD), g/dL		11.8^b^ (0.69)	12.0 (1.58)	11.8 (1.22)
Blood pressure^a^, mean (SD), mmHg	Systolic	138.0 (28.68)	142.4 (20.51)	134.1 (14.78)
	Diastolic	77.6 (15.41)	84.3 (9.20)	75.5 (9.21)
Comorbidities, *n* (%)	Hypertension	11 (92%)	12 (100%)	11 (92%)
	Diabetes	6 (50%)	6 (50%)	7 (58%)
	Cardiovascular disease^c^	2 (17%)	7 (58%)	3 (25%)
	History of extremity amputation	4 (33%)	2 (17%)	3 (25%)
	Cancer	1 (8%)	0 (0%)	0 (0%)

*y* years, *kg* kilogram, *s* seconds, K/µL = 10^3^/microliter, *g/dL* grams/deciliter, *mmHg* millimeters mercury.

^a^Value at screening.

^b^*n* = 11 patients.

^c^Includes cardiac bypass, cardiac stent, cerebral aneurysm, congestive heart failure, coronary artery disease, myocardial infarction, stroke, and transient ischemic attack.

### Safety

There were no deaths or patient discontinuation due to adverse events (AE) in this study. Two patients experienced unrelated serious adverse events (SAE) in this study: one patient experienced toxicity to baclofen and one patient experienced symptomatic hyperkalemia prior to study drug administration. Treatment-emergent adverse events (TEAE) were minimally reported, with only 3 TEAE experienced by 2/36 (6%) patients (Table 2). One patient experienced muscle spasms and toxicity related to baclofen administration 12 h following the 1.5 µg/kg dose of AB002 (as noted above), and one patient experienced hypotension 2.5 h following placebo. No patients experienced clinically relevant bleeding while on study and time to hemostasis at the vascular access site was not changed in patients receiving AB002 compared with their own baseline or placebo (Table 3). Blood coagulation parameters (thrombin time [TT], prothrombin time [PT], and activated partial thromboplastin time [aPTT]) remained unchanged in each patient when baseline (day −8) was compared to post-dose (day 3) (Supplementary Table 2), and there was no evidence of an anti-drug antibody response resulting in the development of new cross-reacting antibodies to either AB002 or purified human thrombin.

**Table 2:** Treatment-Emergent Adverse Events

Variable	Overall (*n* = 36)	Placebo Pooled (*n* = 12)	AB002 1.5 µg/kg (*n* = 12)	AB002 3 µg/kg (*n* = 12)
Patients With TEAE	2 (6%)	1 (8%)	1 (8%)	0 (0%)
Patients Without TEAE	34 (94%)	11 (92%)	11 (92%)	12 (100%)
Number of TEAEs	3	1	2	0
AE^a^ / Severity^b^ / Relatedness		Hypotension / Grade 2 / Unlikely	Muscle spasms / Grade 1 / Unrelated Baclofen toxicity / Grade 3 / Unrelated	

^a^AEs are classified according to MedDRA version 21.1.

^b^Severity: Grade 1 = mild, Grade 2 = Moderate, Grade 3 = Severe, Grade 4 = Potentially life-threatening.

**Table 3:** Time to Hemostasis at Vascular Access Site

	All Days	Non-Dosing Days^a^			Day of Dosing		
	Overall^b^ (*n* = 34)	Placebo^b^ (*n* = 10)	AB002 1.5 µg/kg (*n* = 12)	AB002 3 µg/kg (*n* = 12)	Placebo^b^ (*n* = 10)	AB002 1.5 µg/kg (*n* = 12)	AB002 3 µg/kg (*n* = 12)
Assessments > 10 min (relative frequency)^c^	17/169 (10%)	3/40 (8%)	8/47^d^ (17%)	2/48 (4%)	3/10 (30%)	1/12 (8%)	0/12 (0%)

^a^Assessments performed on non-dosing days (days −7, −5, −2, 3) were combined.

^b^Two patients had chest catheters and were not assessed for time to hemostasis.

^c^Defined as the number of events in which the time to hemostasis assessment was greater than 10 minutes divided by the total number of assessments per category and expressed as a percentage for comparison across treatment groups.

^d^One assessment was not performed because patient in the 1.5 µg/kg treatment group received hemodialysis off-site on day −2.

There were no clinically notable shifts from baseline to post-dose in coagulation parameters (Supplementary Table 2), serum chemistry (Supplementary Data 2), or hematology (Supplementary Data 3). Mean vital sign parameters and mean ECG parameters remained within normal limits following all treatments at all post-dose time points and changes from baseline were minimal.

### Efficacy

**Fig. 2:**
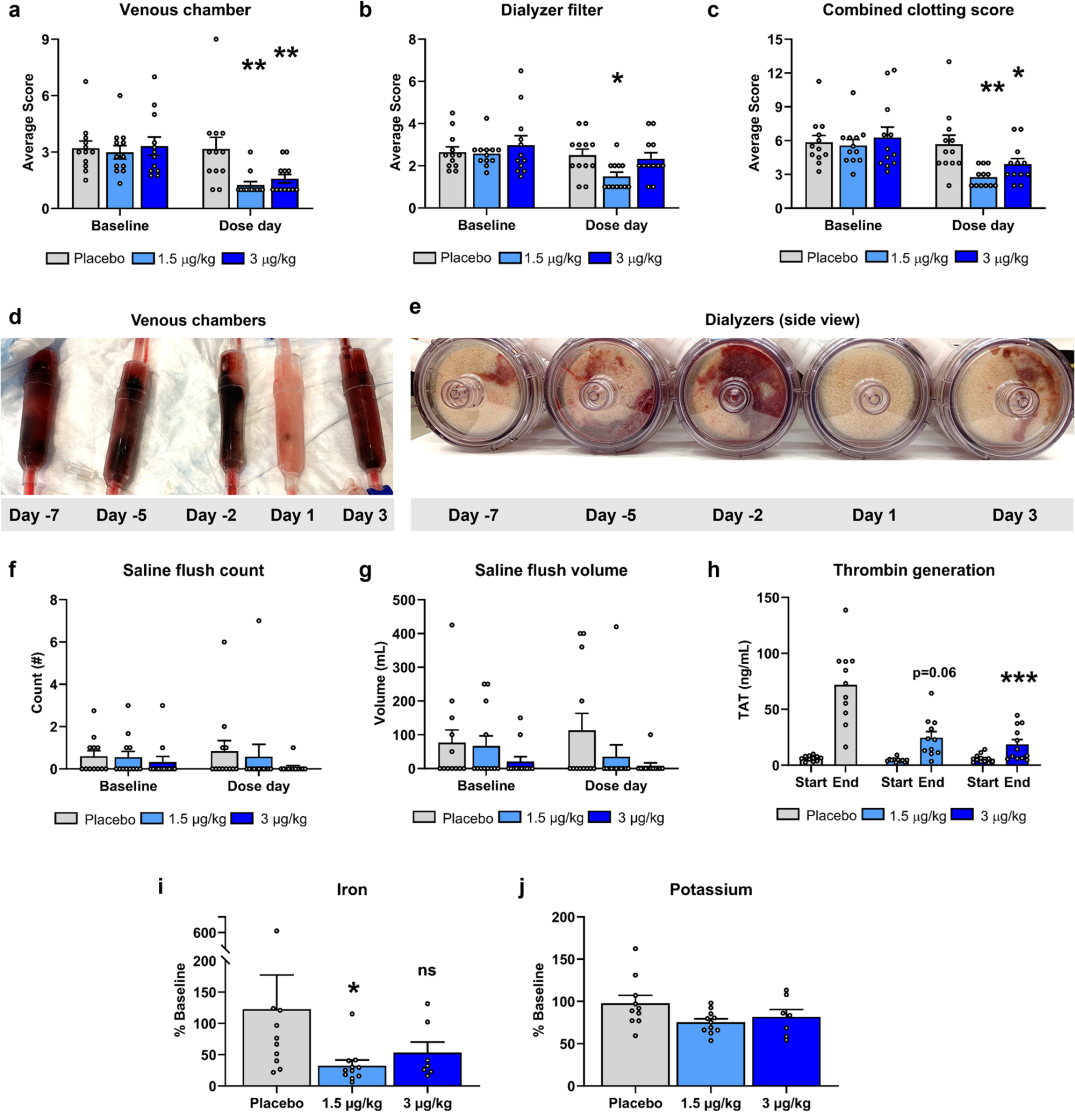
Circuit clotting, thrombin generation, and blood entrapment. Severity of clotting within the circuit at the end of hemodialysis was evaluated in the venous chamber and dialyzer filter for each session using a numeric visual score to generate a mean clotting score at baseline (non-dosing days −7, −5, −2, 3) and dose day (day 1) for each patient. The mean clotting scores from the venous chamber (**a**), dialyzer filter (**b**), and the combined score of both (**c**) are presented by treatment group (*n* = 12 patients for placebo [gray bars], *n* = 12 for 1.5 µg/kg [light blue bars], and *n* = 12 for 3 µg/kg [dark blue bars] groups). Data represent means ± SEM; *p < 0.05 and **p < 0.01 comparing change from baseline scores vs placebo. In (**d**, **e**), representative photographs of a series of venous chambers and side view of dialyzer filters from two separate patients that received AB002 on day 1 are shown. The number (**f**) and total volume (**g**) of hemodialysis circuit saline flushes required to maintain circuit patency on non-dosing days were recorded to generate an average value at baseline and were compared to the means for the day of dosing for each treatment group. Thrombin-antithrombin (TAT) complexes (**h**) were measured in patient plasma samples at the start and end of hemodialysis to examine the effect of AB002 on systemic thrombin generation. *n* = 11 for placebo, *n* = 11 for 1.5 µg/kg, and *n* = 12 for 3 µg/kg; data represent means ± SEM, ***p < 0.001 comparing change vs placebo. AB002 reduced iron (**i**) and potassium (**j**) retained with**i**n the hemodialysis cartridge due to blood clotting on study day 1 compared to placebo. *n* = 10 for placebo, *n* = 11 for 1.5 µg/kg, and *n* = 7 for 3 µg/kg, data represent means ± SEM, *p < 0.05 vs placebo. Exact *p*-values are provided in Supplementary Data 1.

No differences in clot severity scores were observed between patients of any groups on non-dosing days (baseline) for either the venous chamber (placebo group: 3.2 [0.38], 1.5 µg/kg group: 3.0 [0.36], 3.0 µg/kg group: 3.3 [0.49]) or dialyzer filter (placebo group: 2.6 [0.25], 1.5 µg/kg group: 2.6 [0.18], 3.0 µg/kg group: 3.0 [0.44]). For patients receiving either dose of AB002, mean [SEM] clot severity scores for the venous chamber (1.5 µg/kg: 1.3 [0.18], 3.0 µg/kg: 1.6 [0.23]) were significantly reduced from baseline compared to placebo (3.2 [0.61]) (both *p* = 0.009). In the dialyzer filter, mean [SEM] clot severity scores (1.5 µg/kg: 1.5 [0.19], 3.0 µg/kg: 2.3 [0.28]) were reduced from baseline compared to placebo (2.5 [0.29]), but only significantly so at the 1.5 µg/kg dose level (*p* = 0.039) (Fig. 2a–e).

A total of 179 hemodialysis sessions were evaluated to determine the effect of AB002 on circuit changeouts. In sessions in which AB002 was not administered (all treatment arms), circuit changeouts occurred approximately 14% (21/155) of the time, which is similar to rates reported elsewhere^17,22,23^ (Table 4). By contrast, circuit changeout was required in 4% (1/24) of the 24 patients that received either dose of AB002.

**Table 4:** Circuit Changeouts by Treatment

	Treatment					
	Placebo		AB002 1.5 µg/kg		AB002 3 µg/kg	
	Non-Dosing	Dosing	Non-Dosing	Dosing	Non-Dosing	Dosing
Changeouts	5/48 (10%)	1/12 (8%)	6/47 (13%)	0/12 (0%)	9/48 (19%)	1/12 (8%)

Among the 36 patients in this study, saline flushes were utilized in 13 patients, with 2 patients requiring saline flushes for every hemodialysis session. Saline flush frequency and volume were lower in patients that received AB002, compared to placebo (Fig. 2f, g and Supplementary Table 3).

To assess thrombin generation during hemodialysis sessions, systemic thrombin-antithrombin (TAT) levels were measured in plasma samples collected 1 hour prior to hemodialysis and again at the end of hemodialysis. Pre-dialysis TAT levels were 6.1 ng/mL, 4.6 ng/mL and 5.9 ng/mL in the placebo, 1.5 µg/kg, and 3 µg/kg AB002 groups, respectively (Fig. 2h). On day of dosing, in patients given placebo, TAT levels measured at the end of hemodialysis were 71.8 ng/mL, approximately a 12-fold increase over starting values. By contrast, AB002 attenuated TAT generation compared with placebo (3-fold increase over starting values in 3 µg/kg group (18.7 ng/mL), *p* = 0.0001; 6-fold increase over starting values in 1.5 µg/kg group (24.7 ng/mL), *p* = 0.062), translating to a maximum reduction of 74% at the 3 µg/kg dose level. Hemodialysis-induced TAT generation was not significantly different across groups on non-dosing days (Supplementary Data 1).

The entrapment of blood within the dialyzer cartridge revealed a notable difference between the AB002 treatment group and the placebo group. Both dose levels numerically reduced potassium and iron entrapment within the dialyzers, with the 1.5 µg/kg dose level reaching statistical significance for iron (*p* = 0.031) (Fig. 2i). Potassium and iron values were not significantly different across treatment groups on non-dosing days (Supplementary Data 1). Taken together, these data suggest that AB002 treatment effectively reduced hemodialysis-associated clotting and thrombus accumulation within the circuit.

### Pharmacodynamics

**Fig. 3:**
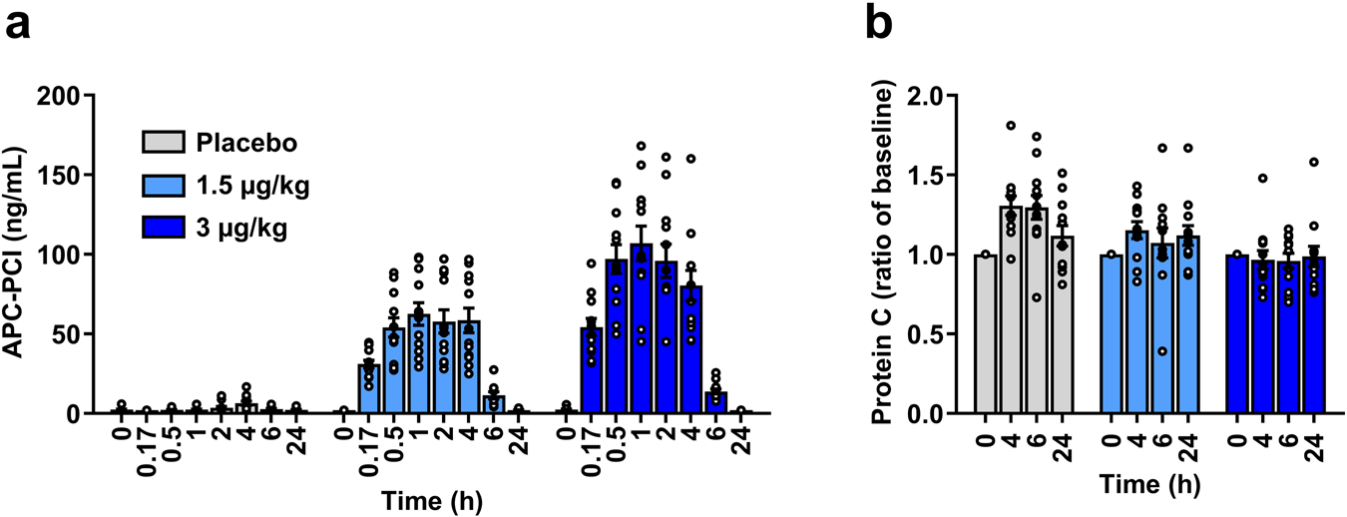
Pharmacodynamic evaluation of AB002 drug exposure. Patients were administered a single dose of AB002 at 1.5 µg/kg or 3 µg/kg, or placebo. The surrogate marker of drug exposure, APC-PCI **(a)**, was evaluated in patient plasma samples on study day 1 through 24 h post-dose. Circulating protein C levels **(b)** were evaluated on study day 1 through 24 h post-dose. Data were normalized to pre-dose values as a ratio of baseline. *n* = 12 for placebo (gray bars), *n* = 12 for 1.5 µg/kg (light blue bars), and *n* = 12 for 3 µg/kg (dark blue bars); data represent means ± SEM. Exact *p*-values are provided in Supplementary Data 1.

On the day of dosing, mean (SD) aPTT values measured at the end of hemodialysis were 32.3 (6.04), 38.7 (7.24), and 33.9 (4.82) seconds in the placebo, 1.5 µg/kg AB002, and 3 µg/kg AB002 groups, respectively. As a marker for APC generation, APC-PCI complex levels were evaluated in plasma. Average APC-PCI concentrations increased rapidly with AB002 dosing (Fig. 3a). Peak mean concentrations were observed at 1 h of infusion and remained measurable in all patients up to 6 h post-dose, returning below the limit of quantitation by 24 h post-dose. In patients who received placebo, circulating protein C levels measured at the end of hemodialysis were increased relative to baseline levels and remained elevated until ~24 h later (Fig. 3b). By contrast, relative protein C levels remained closer to baseline in patients who received either dose of AB002.

### Hemodialysis efficiency

For all patients, hemodialysis parameters were set to a blood flow rate of 350 mL/min and a dialysate flow rate of 500 mL/min. Mean potassium and blood urea nitrogen (BUN) values decreased following hemodialysis (Supplementary Table 4) with no differences across or within treatment groups. Mean urea reduction ratio (URR), Kt/V values, and ultrafiltration volume were not different across or within treatment groups.

## Discussion

As far as we are aware, this phase 2 proof-of-concept clinical trial is the first to assess the safety and efficacy of therapeutic protein C activation. AB002, administered during hemodialysis, appeared to be safe and well-tolerated, with no treatment-related complications or adverse events. Clinically detected and relevant thrombosis or bleeding did not occur in any patient and the time to hemostasis at the vascular access site did not appear to be affected by AB002. There was no evidence of AB002-induced anti-drug or anti-thrombin antibodies at 14 days post-dose. Importantly, AB002 was able to safely limit hemodialysis-associated clotting in a medically complex ESRD patient population, who are at an increased risk of both bleeding and thrombosis^24,25^.

The pharmacologic effect of AB002 infusion, the generation of APC, was confirmed by the dose-dependent increase in APC-PCI complexes in all patients who received the drug. Plasma protein C levels appeared to be transiently elevated after hemodialysis, which may be attributable to hemoconcentration. This elevation in protein C following hemodialysis was not observed in patients treated with AB002, raising the possibility that AB002 may have caused some consumption of the circulating protein C pool as a result of protein C activation and elimination. This explanation is consistent with observations in the phase 1 trial, in which there was a small temporal reduction in plasma protein C levels that returned to baseline levels within 24 hours following bolus administration of AB002 to healthy volunteers^8^. Consistent with the anticoagulant/antithrombotic effects of endogenous APC generation, coagulation activation resulting from the hemodialysis procedure was markedly reduced in AB002-treated patients, as evidenced by reduced systemic plasma TAT levels.

The exploratory efficacy endpoints in this study suggest that AB002 reduced circuit clotting during heparin-free hemodialysis. Compared to placebo, the severity of macrothrombi in the dialyzer circuit, assessed via visual scoring, was significantly reduced in patients when they received AB002. Consistent with clot severity results, blood clot derived iron present in the dialyzer filters was reduced from baseline in patients that received AB002 (reaching significance at the 1.5 µg/kg dose level) compared to placebo. The frequency of circuit changeouts was noticeably less in patients that received AB002. Lastly, saline priming frequency and volume were decreased in patients that received AB002, compared to placebo.

The doses selected for this study were based upon safety data from the phase 1 study and preclinical efficacy studies demonstrating that AB002 rapidly interrupts experimental arterial-type thrombus propagation at bolus doses of 2 µg/kg^8^. In the present study, we aimed to achieve a sustained antithrombotic effect for the duration of a 4-hour hemodialysis session, thus we selected doses that were observed to be effective in that model. Although two dose levels were evaluated in this study, it appears that we may have reached the limit for clinical efficacy at the 1.5 µg/kg dose level, since we did not see any additional effect on clotting or circuit changeouts at the higher dose level of 3 µg/kg, despite an increase in the biomarker APC-PCI and a decrease in TAT generation.

This trial was not intended to investigate AB002 in comparison to heparin, as the intended initial target populations are those patients who are not suitable for intradialytic heparin use. However, aside from the increased bleeding risk associated with systemic use during hemodialysis^26^, heparin is known to have heterogenous effects that complicate dosing in ESRD patients, including bone mineral disease, hyperkalemia, and hypertriglyceridemia^27^. Clinical interest in heparin alternatives has understandably been focused on patient populations with known heparin intolerance or elevated bleeding risk, but safe non-heparin alternatives could displace heparin if clinical outcomes, such as reduction in morbidity and mortality compared to heparin, justify such a move.

Based on non-clinical and clinical data, administration of very low, yet antithrombotic doses of AB002 ( < 4 µg/kg, intravenous bolus or infusion) does not appear to alter any standard laboratory markers of hemostasis, such as coagulation, bleeding times, or platelet aggregation^8^. This is fundamentally different from the changes induced by other antithrombotic agents, such as platelet antagonists and anticoagulants, including infused recombinant APC. We thus contemplate that in addition to ESRD patients who cannot receive heparin due to increased risk of bleeding, AB002 may be suitable as a non-heparin option in patients with a history of heparin-induced thrombocytopenia (HIT). HIT is a rare, but life-threatening complication of heparin exposure that results in disseminated or peripheral blood clots, hypercoagulability, and thrombocytopenia. Repeated heparin exposure in the course of maintenance hemodialysis is of particular concern for the development of HIT^28^.

The limitations of this study include the small patient number, limited racial, ethnic, and female representation, and single-dose administration, which preclude a conclusion of whether AB002 employed as a multiple dose regimen can achieve safe and functional clot prevention. This study was conducted at a single site and reflects the demographic makeup of the region. While the short half-life of AB002 is advantageous for use during hemodialysis, it restricted our ability to assess long term effects of repeat-dose treatment on factors such as anemia or chronic inflammation, which contribute to morbidity and mortality associated with chronic hemodialysis in ESRD. Given the anti-inflammatory and cytoprotective effects of recombinant APC administration in disease models^29–32^, including in ischemic stroke^33,34^, it is conceivable that AB002 may also impart beneficial effects on chronic inflammation or anemia in this patient population. Additional studies in which AB002 is administered repeatedly during heparin-free hemodialysis will be critical to testing this hypothesis.

In conclusion, AB002 was well-tolerated and reduced thrombus accumulation in the hemodialysis circuit without observable hemostasis impairment in ESRD patients undergoing heparin-free hemodialysis. Based on a vast body of preclinical data and the compelling clinical study results outlined here, protein C activation may be useful in multiple acute thrombotic and thrombo-inflammatory indications. Indeed, modulating the protein C system via AB002 or related precursor enzymes has been shown in animal models to limit inflammation, reduce thrombus initiation, and to promote thrombolysis, without increased bleeding^5,6,8,35–40^. Thus, AB002 may be useful not only for thrombo-prophylactic indications such as during hemodialysis, but also for acute clotting conditions that include stroke, heart attack, or pulmonary embolism, where there is a compelling need for safer antithrombotics. Overall, the results of this study support further clinical evaluation of AB002 as a drug candidate to safely treat or prevent thrombus development or blood loss due to device-initiated clotting in patients with elevated bleeding risk, including ESRD patients on chronic hemodialysis.

### Supplementary information

Supplementary Information

Description of Additional Supplementary Files

Supplementary Data 1

Supplementary Data 2

Supplementary Data 3

Reporting Summary

## Data Availability

The numerical data underlying Figs. 2 and 3 can be found in Supplementary Data 1. Source data for patient coagulation values and hemodialysis efficiency can be found in Supplementary Tables 2 and 4. Source data for serum chemistry and hematology values can be found in Supplementary Data 2 and 3. To ensure patient confidentiality, deidentified individual participant data underlying the reported results will be made available upon publication for a period of 5 years. Proposals for access should be sent to: norah.verbout@aronorabio.com.
