# Supplementary material for: Safety and tolerability of the protein C activator AB002 in end-stage renal disease patients on hemodialysis: a randomized phase 2 trial
Source: Commun Med (Lond). 2024 Jul 26;4:153. doi: 10.1038/s43856-024-00575-y (PMC11282208; doi:10.1038/s43856-024-00575-y)
Supplement: Supplementary file 1 — Supplementary Information [file 43856_2024_575_MOESM1_ESM.pdf]

Supplementary Information

Supplementary Table 1. Visual score assessment of clotting in the hemodialysis circuit

Dialyzer Filter	
Score	Description
1	No clotting/clean dialyzer or few blood streaks affecting less than 5% of the fibers seen at the surface of the dialyzer
2	Blood streaks affecting more than 5% but less than 25% of the fibers seen at the surface of the dialyzer
3	Blood streaks affecting more than 25% but less than 50% of the fibers seen at the surface of the dialyzer
4	Blood streaks affecting more than 50% but less than 75% of the fibers seen at the surface of the dialyzer
5	Blood streaks affecting more than 75% of the fibers seen at the surface of the dialyzer
--	Complete occlusion, coagulated filter (treatment cannot continue without replacement of dialyzer)*
Venous Chamber	
Score	Description
1	No detectable clotting
2	Presence of fibrous ring or minimum clot affecting less than 5% of chamber space
3	Clot formation, affecting more than 5% but less than 25% of chamber space
4	Clot formation, affecting more than 25% but less than 50% of chamber space
5	Clot formation, affecting more than 50% but less than 75% of chamber space
6	Clot formation, affecting more than 75% of chamber space
--	Complete occlusion of chamber*
* occlusions were recorded but not scored	

Supplementary Table 2. Summary of coagulation measurements at baseline and postdose

Laboratory Test (units)	Time Point	Statistic	Treatment		
			Placebo (pooled) (n=12)	AB002 1.5 µg/kg (n=12)	AB002 3 µg/kg (n=12)
Fibrinogen (mg/dL)	Baseline	Mean	353.5	355.5^	383.7
		SD	82.81	66.80^	67.76
	Postdose	Mean	342.3	333.8	371.5^
		SD	89.23	76.37	68.28^
Prothrombin Time/INR (Ratio)	Baseline	Mean	1.05	1.08	1.04
		SD	0.100	0.147	0.079
	Postdose	Mean	1.05	1.05	1.05^
		SD	0.090	0.124	0.121^
Prothrombin Time (sec)	Baseline	Mean	11.78	12.14	11.88
		SD	1.050	1.792	1.157
	Postdose	Mean	11.66	11.58	11.92^
		SD	0.989	1.545	1.480^
Thrombin Time (sec)	Baseline	Mean	15.81	15.63	16.13
		SD	1.681	2.236	1.013
	Postdose	Mean	16.19	15.74	16.40^
		SD	1.310	0.948	1.430^
Activated Partial Thromboplastin Time (sec)	Baseline	Mean	33.5	34.73	31.93
		SD	4.940	3.732	3.528
	Postdose	Mean	32.93	33.10	32.16^
		SD	3.326	4.292	2.885^

Baseline defined as day -8
Postdose defined as day 3
^ n=11

Supplementary Table 3. Summary of changes in saline flush count and flush volume

Measure	Treatment		
	Placebo (pooled) (n=6)	AB002 1.5 µg/kg (n=4)	AB002 3 µg/kg (n=3)
% Change in Saline Flush Count	138%	105%	25%
% Change in Saline Volume	147%	53%	40%

The change in flush count and volume are presented as the average change (mean dose day/mean non-dosing days). Not all participants had saline flushes during hemodialysis, thus the data are presented cumulatively and the standard deviation was not calculated.

Supplementary Table 4. Summary of potassium, BUN, URR and Kt/V

Day	Statistic	Treatment														
		Placebo (pooled)					AB002 1.5 µg/kg					AB002 3 µg/kg				
		ΔK (ratio)	ΔBUN (mg/dL)	URR (%)	Kt/V	UF Vol (mL)	ΔK (ratio)	ΔBUN (mg/dL)	URR (%)	Kt/V	UF Vol (mL)	ΔK (ratio)	ΔBUN (mg/dL)	URR (%)	Kt/V	UF Vol (mL)
-7	n	11	11	11	11	12	12	12	12	12	12	11	11	11	11	12
	Mean	0.71	36.9	69.68	1.392	2007	0.78	41.1	69.48	1.411	2985	0.75	39.4	68.16	1.367	2979
	(SD)	(0.064)	(10.13)	(69.68)	(0.2505)	(939.7)	(0.066)	(10.01)	(5.457)	(0.2261)	(1072.1)	(0.091)	(14.92)	(6.419)	(0.2597)	(1387.8)
-5	n	8	7	7	7	12	11	11	11	11	12	12	12	12	12	12
	Mean	0.70	37.4	71.44	1.470	2446	0.72	38.0	70.48	1.436	2875	0.83	35.3	68.43	1.343	2320
	(SD)	(0.068)	(8.70)	(6.975)	(0.2408)	(985.3)	(0.068)	(7.06)	(5.709)	(0.2025)	(1056.7)	(0.337)	(10.72)	(6.646)	(0.2429)	(1278.8)
-2	n	11	11	11	11	12	11	10	10	10	11	11	10	10	10	12
	Mean	0.70	45.2	68.69	1.388	3064	0.75	44.6	68.51	1.372	3035	0.73	44.7	68.62	1.391	2908
	(SD)	(0.099)	(8.45)	(6.849)	(0.2357)	(1046.4)	(0.076)	(7.53)	(7.467)	(0.2657)	(1253.9)	(0.116)	(10.98)	(6.496)	(0.2427)	(1299.3)
1	n	11	11	11		12	12	12	12	12	12	11	11	11	11	12
	Mean	0.68	40.9	69.77	111.418	2603	0.73	41.3	70.63	1.468	2824	1.75	38.6	70.61	1.553	2573
	(SD)	(0.085)	(8.17)	(6.135)	(0.2312)	(778.1)	(0.074)	(10.90)	(6.955)	(0.2542)	(1019.7)	(0.160)	(8.88)	(5.146)	(0.4112)	(1115.3)
3	n	12	12	12	12	12	11	10	10	10	12	12	12	12	12	12
	Mean	0.75	37.9	67.08	1.319	2462	0.72	41.0	69.09	1.381	2363	0.76	35.8	69.21	1.373	2263
	(SD)	(0.087)	(7.89)	(8.132)	(0.2908)	(914.9)	(0.083)	(7.29)	(7.599)	(0.2437)	(1219.9)	(0.070)	(6.82)	(6.391)	(0.2366)	(922.2)

ΔK = Fold change (Post-hemodialysis potassium value) / (Pre-hemodialysis potassium value)
ΔBUN = (Pre-hemodialysis BUN value) – (Post-hemodialysis BUN value)
URR (Urea Reduction Rate) = 100 × (1-Post-hemodialysis BUN/Pre-hemodialysis BUN))
Kt/V = $-\ln(R-(0.008 \times t)) + (4-(3.5 \times R)) \times UF/Wt$
UF Vol = Ultrafiltration volume

Supplementary Methods

Study Protocol

Clinical Protocol

A Phase 2, Randomized, Double-Blind, Placebo-Controlled Study to Assess the Safety and Efficacy of a Single Dose of E-WE Thrombin, Administered During a Regular Hemodialysis Procedure, in Patients with End-Stage Renal Disease on Chronic Hemodialysis

Celerion Project No.: CA26622

Sponsor Project No.: EWE-19-02

US IND No.: 18154

GCP Statement

This study is to be performed in full compliance with the protocol, Good Clinical Practices (GCP), and applicable regulatory requirements. All required study documentation will be archived as required by regulatory authorities.

Confidentiality Statement

This document is confidential. It contains proprietary information of Aronora Inc., and/or Celerion. Any viewing or disclosure of such information that is not authorized in writing by Aronora Inc., and/or Celerion is strictly prohibited. Such information may be used solely for the purpose of reviewing or performing this study.

1. PROTOCOL REVISION HISTORY

<table>
<tr><th>DATE/NAME</th><th>DESCRIPTION</th></tr>
<tr><td>22April2020 by Natacha Benrimoh</td><td>Final Protocol, Amendment 2:

This protocol was amended to update exclusion criterion #9 in order to aid in recruitment. Cut--off values for hemoglobin entry levels is not required for this study’s population (patients with end-stage renal disease). Removal of this criterion will not affect patients safety upon drug administration and will not interfere with study endpoints.

Therefore, the last bullet in Section 11.2 Exclusion Criteria, criterion # 9, “Hemoglobin concentration < 10 g/dL at screening or check-in on Day -8” was removed.

In addition, the protocol clarification dated February 12, 2020 was incoporated in this amendment. In Section 13.5.1 Blood Sampling and Processing and in Section 13.7.1 Protein C Analysis, allowable time deviation window for post-dose blood collections were thus added.</td></tr>
<tr><td>01May2019 by Joelle Batonga</td><td>Final Protocol, Amendment 1:

This protocol was amended at the Sponsor’s request to add sample timepoints for protein C in order to characterize protein C in all hemodialysis sessions. Additional samples will be collected at the start and at the end of hemodialysis on Days -7, -5, -2, and 3. Thus the total number of time points for protein C analysis sample collection will increase from 8 to 16:

In Section 6 – Study Event Flow Chart, collection of blood samples for protein C analysis were added at the start and the end of hemodialysis on Days -7, -5, -2, and 3.

In Section 13.8 - Blood Volume for Study Assessments, Table 3 - Blood Volume during the Study, was amended as follows (deleted text in <s>strikethrough</s> and added text in bold in a condensed version of the table):
<table>
<tr><th>Sample Type</th><th>Number of Time Points</th><th>Approximate Volume per Time Point * (mL)</th><th>Approximate Sample Volume Over Course of Study (mL)</th></tr>
<tr><td>Blood for protein C (ships to Aronora)</td><td><s>8</s>16</td><td>2</td><td><s>16</s>32</td></tr>
<tr><td colspan="3">Total Blood Volume (mL)→</td><td>Up to <s>220</s>236***</td></tr>
</table></td></tr>
<tr><td>15Mar2019 by Joelle Batonga</td><td>Final Protocol</td></tr>
</table>

2. PRINCIPAL INVESTIGATOR AND SPONSOR – SIGNATORIES

A Phase 2, Randomized, Double-Blind, Placebo-Controlled Study to Assess the Safety and Efficacy of a Single Dose of E-WE Thrombin, Administered During a Regular Hemodialysis Procedure, in Patients with End-Stage Renal Disease on Chronic Hemodialysis

SPONSOR: Aronora, Inc.
4640 SW Macadam Avenue, Suite 200A
Portland, Oregon 97239, USA
Tel.: +1 503 964-0250

SPONSOR'S REPRESENTATIVE: Norah Verbout, PhD
Senior Scientist
4640 SW Macadam Avenue, Suite 200A
Portland, Oregon 97239, USA
Tel.: +1 503 964-0250
E-mail: norah.verbout@aronorabio.com

Signature Date

CLINICAL SITE AND PRINCIPAL INVESTIGATOR

Site Address: Orlando Clinical Research Center
5055 S. Orange Avenue
Orlando, Florida. 32809-3017, USA
Tel.: +1 407 240-7878

Signature Date

Printed Name

3. ADDITIONAL KEY CONTACTS FOR THE STUDY

**Sponsor Contact for Serious Adverse Events**	Andras Gruber, MD<br>President and CEO<br>4640 SW Macadam Avenue, Suite 200A<br>Portland, Oregon 97239, USA<br>Tel.: +1 503 530-6842<br>Mobile: +1 503 442-1213<br>Fax: +1 503 389-7330<br>E-mail: andras.gruber@aronorabio.com
**Celerion Protocol Author**	Joelle Batonga, MSc<br>Senior Pharmacokinetic Scientist I,<br>Data Management and Biometrics<br>Celerion<br>100 Alexis-Nihon Boulevard, Suite 360<br>Montreal, Quebec H4M 2N8, Canada<br>Tel.: +1 514 744-8745<br>Fax: +1 514 744-8700<br>E-mail: joelle.batonga@celerion.com
**Bioanalytical Laboratory for APC-PCI Samples**	Celerion<br>621 Rose Street<br>Lincoln, Nebraska 68502, USA<br>Tel.: +1 402 476-2811<br>Fax: +1 402 939-0428
**Shipment Address for Protein C, Total Protein Accumulation and Dialysate Samples**	Aronora, Inc.<br>4640 SW Macadam Avenue, Suite 200A<br>Portland, Oregon 97239, USA<br>Tel.: +1 503 964-0250<br>Fax: +1 503 389-7330
**Immunogenicity Testing Laboratory**	Haemtech Biopharma Services, Inc.<br>57 River Road, Unit 1010<br>Essex Junction, Vermont 05452, USA<br>Tel.: +1 802 878-1777<br>Fax: +1 802 878-1776
**Clinical Laboratory**	Orlando Clinical Research Center<br>5055 S. Orange Avenue<br>Orlando, Florida 32809-3017<br>Tel.: +1 407 240-7878<br>Fax: +1 407 240-9846

**Pharmacodynamic and Statistical Analyses**	Celerion<br>100 Alexis-Nihon Boulevard, Suite 360<br>Montreal, Quebec H4M 2N8, Canada<br>Tel.: +1 514 744-9090<br>Fax: +1 514 744-8700<br>and/or<br>621 Rose Street<br>Lincoln, Nebraska 68502, USA<br>Tel.: +1 402 476-2811<br>Fax: +1 402 476-7598

4. TABLE OF CONTENTS

1. PROTOCOL REVISION HISTORY 2
2. PRINCIPAL INVESTIGATOR AND SPONSOR – SIGNATORIES 3
3. ADDITIONAL KEY CONTACTS FOR THE STUDY 4
4. TABLE OF CONTENTS 6
5. SYNOPSIS 9
6. STUDY EVENTS FLOW CHART 13
7. ABBREVIATIONS 16
8. BACKGROUND AND RATIONALE 19
8.1 Introduction 19
8.2 Background 19
8.2.1 Nonclinical Studies 19
8.2.1.1 Pharmacokinetics 20
8.2.1.2 Pharmacodynamics 20
8.2.1.3 Toxicology 21
8.2.2 Clinical Studies 21
8.2.2.1 Safety Data 22
8.2.2.2 Pharmacodynamic Data 22
8.3 Rationale 24
8.3.1 Purpose of the Study Design 24
8.3.2 Dose Selection 26
8.3.3 Rationale for Pharmacodynamic Markers 26
8.4 Risk/Benefit 26
9. STUDY OBJECTIVES AND ENDPOINTS 28
9.1 Study Objectives 28
9.2 Study Endpoints 28
10. INVESTIGATIONAL PLAN 29
10.1 Overall Study Design and Plan 29
10.1.1 Confinement, Return Visits, and Follow-Up 30
10.1.2 Study Duration 30
10.2 Study Conduct 30
11. STUDY POPULATION 30
11.1 Inclusion Criteria 30
11.2 Exclusion Criteria 31
11.3 Early Termination of Patients from the Study 34
11.4 Study Restrictions 35

11.4.1 Prohibitions and Concomitant Medication 35
11.4.2 Meals 35
11.4.3 Activity 35

12. TREATMENTS 36
12.1 Treatments Administered 36
12.2 Dose Modification 37
12.3 Method of Assigning Patients to Treatment Groups 37
12.4 Blinding 37
12.4.1 Maintenance of Randomization 37
12.4.2 Procedures for Breaking the Blind Prior to Study Completion 37
12.4.2.1 Revealing of Randomization 38
12.4.3 Treatment Compliance 38

13. STUDY ASSESSMENTS AND PROCEDURES 39
13.1 Screening 39
13.2 Safety and Tolerability Assessments 39
13.2.1 Body Weight 39
13.2.2 Physical Examination 39
13.2.3 Vital Signs 40
13.2.4 ECG Monitoring 40
13.2.5 Vascular Access Site Reaction Assessment 40
13.2.6 Bleeding of Access Site 41
13.2.7 Clinical Laboratory Tests 42
13.2.8 Adverse Events 43
13.2.8.1 Adverse Event Definition 43
13.2.8.2 Monitoring 43
13.2.8.3 Reporting 43
13.2.8.4 Serious Adverse Events 45
13.3 Hemodialysis Procedure and Blood Clots in the Dialyzer Cartridge 45
13.4 Immunogenicity 46
13.4.1 Blood Sampling and Processing for Immunogenicity 46
13.4.2 Analytical Method 46
13.5 Pharmacodynamic Assessment – APC-PCI 46
13.5.1 Blood Sampling and Processing 46
13.5.2 Analytical Method 47
13.6 Pharmacodynamic Assessments – Drug Efficacy and Efficiency of HD 47
13.7 Internal Analyses 47
13.7.1 Protein C Analysis 48
13.7.2 Dialysate Analysis 48

13.7.3 Analysis of Protein Accumulation Within the Dialyzer Cartridge 48
13.8 Blood Volume for Study Assessments 49
14. STATISTICAL CONSIDERATIONS 50
14.1 Sample Size Determination 50
14.2 Population for Analyses 50
14.3 Statistical Analyses 50
14.3.1 Safety and Tolerability Analyses 50
14.3.2 Immunogenicity Analyses 51
14.3.3 Pharmacodynamic Analyses 51
15. STUDY ADMINISTRATION 52
15.1 Ethics 52
15.1.1 Institutional Review Board 52
15.1.2 Ethical Conduct of the Study 52
15.1.3 Subject Information and Consent 52
15.1.4 Confidentiality 52
15.2 Termination of the Study 53
15.3 Data Quality Assurance 53
15.4 Direct Access to Source Data/Documents 53
15.5 Drug Supplies, Packaging and Labeling 53
15.6 Data Handling and Record Keeping 54
15.7 Report Format 54
15.8 Protocol Amendments 54
15.9 Publication Policy 54
16. REFERENCES 55

LIST OF TABLES

Table 1. Cohort and Treatment Randomization 29
Table 2. Investigational Products 36
Table 3. Blood Volume during the Study 49

LIST OF FIGURES

Figure 1. APC-PCI Complexes after E-WE Thrombin Administration in Healthy Subjects 23
Figure 2. Coagulation Parameters after E-WE Thrombin Administration in Healthy Subjects 24

5. SYNOPSIS

Compound	E-WE thrombin (AB002)
	Active ingredient: E-WE thrombin (0.1 mg/mL)<br>Buffer: 16.2 mM sodium citrate, 3.8 mM citric acid, 150 mM sodium chloride, 0.1% polysorbate 80, at pH 6
Clinical Indication	Prevention of clot formation in hemodialyzer circuits during hemodialysis (HD)
Study Phase	Phase 2
Study Objectives	The primary objective of this study is:<br>• To assess the safety and tolerability of a single dose of E-WE thrombin infused into the dialysis line during a 4-hour HD procedure in patients with end stage renal disease (ESRD).<br>The secondary objectives of this study are:<br>• To assess the pharmacodynamics (PD) of a single dose of E-WE thrombin infused into the dialysis line during a 4-hour HD procedure in patients with ESRD. Activated protein C/protein C inhibitor complex (APC-PCI) will be used as a surrogate biomarker for drug exposure.<br>• To assess the efficacy of a single dose of E-WE thrombin infused into the dialysis line during a 4-hour HD procedure in patients with ESRD. Efficacy will be assessed by thrombus accumulation within the dialyzer cartridge (evaluated by visual inspection) and measurement of urea removed by 4 hours of dialysis (ratio of volume of fluid completely cleared of urea [Kt/V] and urea reduction ratio [URR]).
Study Design	This is a phase 2, randomized, double-blind, placebo-controlled, single-dose study of E-WE thrombin designed to evaluate the safety and efficacy at 2 dose levels administered to patients with ESRD during HD conducted at one study center in the United States (US).<br><br>Patients will be enrolled in Cohort 1 or Cohort 2; cohorts will be dosed sequentially. Dosing of Cohort 2 will commence following completion of all study events for all patients in Cohort 1. Within each cohort, patients will be randomized to receive active drug or matching placebo as summarized below:

Cohort	Treatment	Active:Placebo	Cohort Size
1	0.5 µg/kg bolus dose administered into the dialysis line immediately followed by a 0.25 µg/kg/hour continuous infusion dose into the dialysis line over 4 hours of E-WE thrombin or matching placebo, for a total of 1.5 µg/kg	2:1	12:6
2	1 µg/kg bolus dose administered into the dialysis line immediately followed by a 0.5 µg/kg/hour continuous infusion dose into the dialysis line over 4 hours of E-WE thrombin or matching placebo, for a total of 3 µg/kg	2:1	12:6

This study includes a screening period of 28 days prior to checking in to the clinical research unit (CRU) on Day -8. From Day -8 (8 days prior to dosing on Day 1) through Day 3, all patients will undergo HD 5 times and will be assessed for all scheduled procedures and endpoints before and after each HD session as per the Study Events Flow Chart (Section 6), using only one type of dialyzer cartridge for each patient.

On Day 1, prior to dosing, patients will undergo baseline measurements followed by initiation of a regular HD procedure, consistently using only one type of dialyzer cartridge for each patient. A single dose of E-WE thrombin or matching placebo will be administered into the dialysis line during the HD procedure. Patients will continue to undergo HD procedures and scheduled assessments from Day 1 through Day 3.

Study assessments will include adverse events (AEs), including HD vascular access site (arteriovenous [AV] fistula or AV graft) reactions, physical examinations, vital sign measurements, 12-lead electrocardiogram (ECGs), clinical laboratory tests, coagulation tests, immunogenicity assessments, and PD blood sampling for APC-PCI and measurement of urea removed by 4 hours of dialysis to be performed throughout the study according to the Study Events Flow Chart (Section 6). On Days -7, -5, -2, 1, and 3, bleeding time from the HD vascular access sites on the AV fistula or AV graft (ladder or buttonhole technique) will be evaluated immediately at the end of the HD session. The dialyzer cartridge will be rinsed, visually inspected and graded against a standardized visual assessment scale at the end of the HD procedure, and photographed on 4 sides.

Additionally, plasma and dialysate samples will be collected and sent to Sponsor along with the used and rinsed dialyzer cartridges for potential internal evaluation of plasma protein C, dialysate urea, and total protein accumulation within the dialyzer cartridge.

	All patients who receive the study drug or matching placebo (including patients who terminate the study early) will return to the CRU on Day 14 (± 2 days) for coagulation tests, immunogenicity sample collection, and to determine if any AE has occurred since the last study visit.
Number of Patients	A total of at least 36 adult male and female (of non-childbearing potential) patients will be enrolled; 18 patients are planned for each cohort.<br>In each cohort, an attempt will be made to enroll at least 50% of females and at least 30% of a race/ethnic minority group. An attempt will also be made to approximately match key characteristics, including sex, average age, and weight between cohorts.
Duration of Participation for Patients	The total planned duration of patient participation is approximately 50 days from screening to follow-up, with CRU confinement from check-in on Day -8 to Day 3. Patients will return to the CRU on Day 14 (± 2 days) for follow-up procedures.
Dosage, Dosage Form, Route, and Dose Regimen:	On Day 1, patients in each cohort will receive a single dose of E-WE thrombin or matching placebo as a bolus followed by an infusion into the dialysis line during a 4-hour HD procedure. Cohort 1 will be completed prior to initiation of Cohort 2.<br>Planned doses will be as follows:<br>Cohort 1: 0.5 µg/kg bolus dose administered into the dialysis line immediately followed by a 0.25 µg/kg/hour continuous infusion dose into the dialysis line over 4 hours of E-WE thrombin or matching placebo, for a total dose of 1.5 µg/kg.<br>Cohort 2: 1 µg/kg bolus dose administered into the dialysis line immediately followed by a 0.5 µg/kg/hour continuous infusion dose into the dialysis line over 4 hours of E-WE thrombin or matching placebo, for a total dose of 3 µg/kg.
Key Assessments	**Safety**<br>Safety assessments will include number and severity of AEs (including HD vascular access site reactions), physical examinations, bleeding time from the HD vascular access sites, vital sign measurements, 12-lead ECGs, clinical laboratory tests, and coagulation tests.<br>Number and severity of AE will be tabulated and summary statistics for the 12-lead ECGs, vital signs, clinical laboratory and coagulation tests may be computed and provided, as deemed clinically appropriate.

	**Pharmacodynamics:**<br>Plasma APC-PCI will be measured as a surrogate biomarker of drug exposure. Efficacy of antithrombotic activity will be evaluated by thrombus accumulation within the dialyzer cartridge (evaluated by visual inspection). Efficiency of HD will be assessed using blood urea nitrogen (BUN) levels before and after dialysis to determine urea removed by 4 hours of dialysis (Kt/V and URR) after E-WE thrombin active treatment versus pre-treatment and placebo.<br>Change from baseline for all PD measurements will be summarized by treatment (2 active dose levels and pooled placebo) and per timepoints using descriptive statistics.<br>**Immunogenicity:**<br>Development of antibodies to E-WE thrombin or wild type (WT) thrombin will be monitored using a multi-tiered approach that will include screening, confirmatory, and if applicable, antibody titer and neutralizing antibody assays.

6. STUDY EVENTS FLOW CHART

**Study Procedures** [a]	**Screening** [b] (Day -37 to Day -9)	**Study Days**																			**FU**[c]
		**Evaluation Period**																			
**Days →**		**-8**	**-7**		**-5**		**-2**		**1**										**2**	**3**	
**HD →**			**HD** [e]		**HD** [e]		**HD** [e]		**HD** [e]											**HD** [e]	
**Hours →**		**C-I** [d]	**start**	**end**	**start**	**end**	**start**	**end**	**P**	**0**	**0.167**	**0.5**	**1**	**2**	**3**	**4**	**6**	**24**	**start**	**end**	
**Administrative Procedures**																					
Informed Consent	X																				
Inclusion/Exclusion Criteria	X	X																			
Medical History	X																				
**Safety Evaluations**																					
Full Physical Examination [f]	X	X																		X [h]	
Abbreviated Physical Examination [f]											X										
Height	X																				
Weight	X	X	X [g]	X [h]	X [g]	X [h]	X [g]	X [h]	X [g]							X [h]			X [g]	X [h]	
12-Lead Safety ECG	X	X										X								X [h]	
Vital Signs (HR, BP, RR, and T)	X	X	X [g]		X [g]		X [g]		X [g]				X						X [g]		
Bleeding Time at Access Site				X [i]		X [i]		X [i]								X [i]				X [i]	
Hem, Serum Chem [j], and UA [k]	X	X																		X	
Coagulation [l]	X	X	X [g]	X [h]	X [g]	X [h]	X [g]	X [h]	X [g]							X [h]			X [g]	X [h]	X
Platelet Count			X [g]	X [h]	X [g]	X [h]	X [g]	X [h]	X [g]							X [h]			X [g]	X [h]	
Serum Pregnancy Test (♀only)	X	X																			
Serum FSH (PMP ♀only)	X																				
Saliva Drug Screen and Saliva/Breath Alcohol Screen	X	X																			
HIV/Hepatitis Screen	X																				
AE Monitoring		X																			X

**Study Procedures** [a]	**Screening** [b] (Day -37 to Day -9)	**Study Days**																			**FU**[c]
		**Evaluation Period**																			
**Days →**		**-8**	**-7**		**-5**		**-2**		**1**									**2**	**3**		
**HD →**			**HD** [e]		**HD** [e]		**HD** [e]		**HD** [e]										**HD** [e]		
**Hours →**		**C-I** [d]	**start**	**end**	**start**	**end**	**start**	**end**	**P**	**0**	**0.167**	**0.5**	**1**	**2**	**3**	**4**	**6**	**24**	**start**	**end**	
ConMeds Monitoring	X	X																			
**Study Drug Administration/ PD/Immunogenicity**																					
E-WE Thrombin/ Matching Placebo Administration [m]										X											
Blood for E-WE Thrombin PD (APC-PCI)									X		X	X	X	X		X	X	X			
Blood for Protein C			X [g]	X [h]	X [g]	X [h]	X [g]	X [h]	X		X	X	X	X		X	X	X	X [g]	X [h]	
Plasma and Serum Immunogenicity									X												X
**Other Procedures**																					
Blood BUN (for Kt/V and URR)			X [g]	X [h]	X [g]	X [h]	X [g]	X [h]	X [g]							X [h]			X [g]	X [h]	
Dialysate Collection			X [n]		X [n]		X [n]					X			X				X [n]		
Ultrafiltration Recorded				X [h]		X [h]		X [h]								X [h]				X [h]	
Visual Assessment Photography and Processing of Dialyzer Membrane				X [o]		X [o]		X [o]								X [o]				X [o]	
Vascular Access Site Reaction Assessment									X							X [p]			X	X [p]	
Confinement in the CRU		X																			
CRU Visits	X																				X

a For details on Procedures, refer to Section 13.

b Within 28 days prior to Day -8.

c All patients who receive the study drug or matching placebo (including patients who terminate the study early) will return to the CRU on Day 14 (± 2 days) for coagulation tests, immunogenicity sample collection, and to determine if any AE has occurred since the last study visit.

d Patients will be admitted to the CRU on Day -8, at the time indicated by the CRU, and will remain confined up to Day 3 or early termination**.**

e During confinement to the CRU, patients will undergo HD 5 times: on Day -7, -5, -2, Day 1, and Day 3. For each patient, the HD will last approximately 4 hours as per patient standard schedule which will be the same throughout the study.

f Symptom-driven physical examination may be performed at other times, at the PI's or designee's discretion.

g To be performed within 1 hour prior to the start of HD.

h To be performed at the end of HD.

i The HD vascular access sites (AV fistula or AV graft puncture sites) are to be evaluated immediately after the end of HD and removal of the needles from the fistula or graft for bleeding cessation or other reactions.

j Samples for serum chemistry will be obtained following a fast of at least 4 hours, however, in case of dropouts or rechecks, patients may not have fasted for 4 hours prior to the serum chemistry sample is taken.

k For anuric patients, UA may not be performed.

l Coagulation parameters include aPTT, PT/INR, fibrinogen, and TT.

m A single dose of E-WE thrombin or matching placebo will be administered into the dialysis line according to the randomization schedule, upon initiation of the HD procedure. The dose will be administered as a bolus dose immediately followed by a continuous infusion over 4 hours. Hour 0 will correspond to the beginning of E-WE thrombin bolus administration.

n The dialysate sample will be taken at 30 minutes and at 3 hours after the start of HD. The dialysate will be frozen and sent to Aronora.

o To be performed when the dialyzer cartridge is removed and collected at the end of HD.

p To be performed within approximately 20 minutes after completion of HD.

Abbreviations: ♀ = Females, AE = Adverse events, APC-PCI = activated protein C/protein C inhibitor complex, aPTT = activated partial thromboplastin time, BUN = Blood urea nitrogen, BP = Blood pressure, C-I = Check-in, Chem = Chemistry, ConMeds = Concomitant medication, CRU = Clinical research unit, ECG = Electrocardiogram, FSH = Follicle-stimulating hormone, FU = Follow-up, Hem = Hematology, HD = Hemodialysis, HIV = Human immunodeficiency virus, HR = Heart rate, INR = international normalized ratio, Kt/V = ratio of volume of fluid completely cleared of urea, P = Predose, PD = Pharmacodynamic, PI = Principal Investigator, PMP = Postmenopausal, PT = prothrombin time, RR = Respiratory rate, T = Temperature, TT = thrombin time, UA = Urinalysis, URR = Urea reduction ratio.

7. ABBREVIATIONS

µg	Microgram
ADA	Anti-drug antibodies
AE	Adverse event
APC	Activated protein C
APC-PCI	Activated protein C with protein C inhibitor
aPTT	Activated partial thromboplastin time
ALT	Alanine aminotransferase
AST	Aspartate aminotransferase
AV	Arteriovenous
BMI	Body mass index
bpm	Beats per minute
BUN	Blood urea nitrogen
°C	Degrees Celsius
CFR	Code of Federal Regulations
cm	Centimeter
CRF	Case report form
CRU	Clinical Research Unit
CS	Clinically significant
ECG	Electrocardiogram
ESRD	End stage renal disease
FDA	Food and Drug Administration
FIH	First in human
FSH	Follicle-stimulating hormone
FU	Follow-up
g	Gram
GCP	Good clinical practice
GLP	Good laboratory practice
HBsAg	Hepatitis B surface antigen
hCG	Human chorionic gonadotropin
HCV	Hepatitis C virus

HD	Hemodialysis
HIT	Heparin-induced thrombocytopenia
HIV	Human immunodeficiency virus
IB	Investigator's brochure
ICF	Informed consent form
ICH	International Council on Harmonization
IND	Investigational New Drug
INR	International normalized ratio
IRB	Institutional Review Board
IV	Intravenous
kg	Kilogram
Kt/V	Ratio of volume of fluid completely cleared of urea
m^2	Meters squared
MedDRA®	Medical Dictionary for Regulatory Activities®
mg	Milligram
mL	Milliliter
mmHg	Millimeter of mercury
msec	Millisecond
n	Sample size
No.	Number
ng	Nanogram
NOAEL	No observed adverse effect level
oz.	Ounce
PD	Pharmacodynamic(s)
PI	Principal Investigator
PK	Pharmacokinetic(s)
PT	Prothrombin time
QA	Quality assurance
QTcF	Corrected QT interval using Fridericia's correction
SAE	Serious adverse event
SAP	Statistical analysis plan
SRC	Safety Review Committee

TEAE	Treatment-emergent adverse event
TT	Thrombin time
ULN	Upper limit of normal
URR	Urea reduction ratio
US	United States
USA	United States of America
WT	Wild type

8. BACKGROUND AND RATIONALE

8.1 Introduction

E-WE thrombin (AB002) is a recombinant antithrombotic protein C activator enzyme analog of human alpha thrombin. Intravenously injected E-WE thrombin generates endogenous activated protein C (APC) on intravascular cell surfaces and does not change fibrinogen or platelet count in healthy adult subjects while at its intended pharmacological doses. Due to avid binding of E-WE thrombin to thrombin receptors, predominantly thrombomodulin and GPIb on cells, therapeutic doses of the free (unbound) active enzyme are likely cleared from the circulation in seconds to minutes. Free E-WE thrombin, if any, that is not associated with a thrombin receptor after the minimum effective dose, is unlikely to exhibit measurable pharmacological activity.

E-WE thrombin was developed by Aronora, Inc. under the product identification number AB002 and is intended for single dose administration into the blood circulation through a venous access. Proposed indications for E-WE thrombin include arterial thrombosis and thromboembolism, including those associated with acute cerebrovascular accidents, acute coronary syndrome and other severe acute thrombus formation-associated ischemic events. Additional indications may include peri-interventional and extracorporeal organ support-associated thromboprophylaxis, and temporal antiinflammatory treatment.

A brief overview of available information regarding AB002 follows below. Details can be found in the E-WE thrombin, AB002 Investigator's Brochure of 25 February 2019 [1].

8.2 Background

8.2.1 Nonclinical Studies

The molecular mechanism by which E-WE thrombin exerts its antithrombotic activity has been proposed to involve the selective activation of surface receptor-bound endogenous protein C under intravascular shear flow. Experimental data suggest that antithrombotic treatment with E-WE thrombin could utilize an endogenous targeting system via cell-surface associated thrombin receptors including, among others, thrombomodulin and glycoprotein Ib [2], which are variably expressed on the luminal surfaces of blood vessels, platelets, and other cells. One of the putative mechanisms of action, *in vivo*, is binding of E-WE thrombin to circulating platelets, which are expected to deliver and concentrate E-WE thrombin on the thrombus, which is the site of desired pharmacological activity. At the thrombus interface, E-WE thrombin cleaves receptor-associated protein C, generating the potent endogenous antithrombotic and antiinflammatory enzyme APC. Unlike antithrombin, a direct inhibitor of enzymatic coagulation factors, endogenous APC cleaves and inactivates co-localized cofactors Va and VIIIa, thereby downregulating prothrombotic but not preventing hemostatic thrombin generation. Both endogenous and infused exogenous APC are capable of interrupting natural and experimental thrombogenesis, however, without the risk of paralyzing hemostasis. In fact, gene silencing or knockdown of factor V and/or VIII results in viable, though hemophilic animals. Complete factor VIII deficiency in people or animals increases the risk of bleeding and causes a bleeding disorder (hemophilia), but is generally compatible with life. Low levels of factor V and factor VIII also increase the risk of bleeding,

but to a lesser degree. By contrast, complete factor X, VII or II deficiencies are embryonic-lethal. Natural endogenous APC has not been shown to completely block factor VIII and factor V activity, and even high dose exogenous recombinant APC infusion has not been consistently linked to bleeding in animal models or in humans. Furthermore, animal experiments and in vitro studies suggest that APC (predominantly protein C receptor bound) exerts additional protective effects by upregulating cytoprotective mechanisms and promoting antiapoptotic and antiinflammatory signaling through cleavage of transmembrane receptors, such as protease activated receptors 1 and 3.

In both mice and primates, E-WE thrombin administration has antithrombotic effects at very low doses that do not have notable antihemostatic activity. The endogenous APC that escapes the surface at low E-WE thrombin doses does not increase clotting and bleeding times outside the normal range. The efficacy of E-WE thrombin has been established in several experimental models, including a mouse model of stroke [3], a mouse model of myocardial ischemia and reperfusion, and a well-established model of vascular graft thrombosis in baboons [4], alone and in combination with fibrinolysis. Importantly, while treatment with E-WE thrombin was efficacious in these experimental models at its minimum effective doses, hemostasis markers (clotting times and bleeding times) remained within the “clinically insignificant” range. These data suggest that, in contrast to currently marketed antithrombotics, most of which target essential elements of the hemostatic coagulation cascade in the fluid phase, E-WE thrombin may preferentially target thrombi without notably increasing bleeding after injury.

8.2.1.1 Pharmacokinetics

Limited pharmacokinetic (PK) studies with E-WE thrombin have been performed in cynomolgus monkeys and in baboons. The PK assay used to detect active E-WE thrombin was only sensitive enough to detect plasma E-WE thrombin following high doses of E-WE thrombin (> 10 μg/kg). The observed PK profile in these animals closely mirrored the drug exposure PD biomarker assays (APC-PCI and activated partial thromboplastin time [aPTT]), which were able to detect the magnitude and duration of action of E-WE thrombin. In blood, E-WE thrombin is inhibited by enzymatic interaction with serine protease inhibitors, and some of these reactions are accelerated by heparin. Therefore, it has been technically challenging to develop an ultrasensitive assay for the residual or trace free E-WE thrombin molecules that may have avoided binding or inhibition and remain circulating in plasma. Free E-WE thrombin does not exert demonstrable pharmacological, procoagulant, or platelet activating effects in the fluid phase.

8.2.1.2 Pharmacodynamics

Injection of E-WE thrombin results in the temporal and selective generation of endogenous APC and subsequent escape of some of this endogenous APC into the blood circulation. In vitro, in the presence of phospholipid membranes, APC inactivates factor Va and factor VIIIa, resulting in a temporal prolongation of the aPTT that is observed at higher doses of E-WE thrombin. The activity of APC is regulated in part through its enzymatic interactions with serine protease inhibitors to form inactive enzyme inhibitor complexes, primarily APC-PCI and APC-antitrypsin complexes. The half-life of exogenous recombinant APC is 15 to 25 minutes in blood, and data from baboons suggest that the half-life of endogenous

APC is comparable. The rate of APC inhibition by PCI in plasma is significantly accelerated in the presence of heparin, and the inactive APC-PCI complex is reported to have a half-life of 20 minutes in the circulation.

In preclinical Study Report No. AB002.4, the APC-PCI complex was evaluated as a biomarker for E-WE thrombin activity in a baboon. The systemic effect of an intravenous (IV) bolus of E-WE thrombin (0.75 to 10 µg/kg) on APC-PCI complex formation and corresponding aPTT was evaluated over 2 hours. Injection of E-WE thrombin caused a dose-dependent increase in APC-PCI plasma levels. Elevated levels of APC-PCI were present between 5 and 120 minutes post-administration at all four dose levels, with peak APC-PCI levels observed approximately 30 minutes after E-WE thrombin injection. By 120 minutes post-injection, APC-PCI values were trending toward baseline.

In the same baboon, aPTT prolongation was also evaluated. E-WE thrombin caused a dose-dependent transient increase in the aPTT at all four dose levels, peaking at approximately 15 to 30 minutes post-administration, with peak prolongation ranging from approximately 1.2-fold to 1.8-fold over baseline value. aPTT returned to baseline or reference range by 120 minutes at all doses tested except the highest dose. The duration and magnitude of the aPTT elevation in response to E-WE thrombin correlated with plasma APC-PCI concentrations and the fold increase in aPTT prolongation significantly correlated with APC-PCI concentration:

($R^2 = 0.85$, $p \leq 0.001$ by linear regression).

The APC-PCI profile of E-WE thrombin was also evaluated in a Good Laboratory Practice (GLP) single dose-acute study in cynomolgus monkeys. Administration of E-WE thrombin to cynomolgus monkeys (12.5, 25, 50 µg/kg) caused a temporal increase in the concentration of APC-PCI complexes, in a dose-dependent manner. In addition, the anticipated minimal to modest temporal PD effect of aPTT prolongation occurred in all test article dose groups. APTT values were within the reference range 24 hours later.

8.2.1.3 Toxicology

The toxicity profile of E-WE thrombin was evaluated in two GLP single-dose acute toxicity studies in Sprague-Dawley rats and cynomolgus monkeys. In rats, a single dose of E-WE thrombin was administered at doses of 0, 30, 100, and 300 µg/kg and animals were evaluated for 3 days (main study) and 29 days (recovery group) after dosing. In cynomolgus monkeys, a single dose of E-WE thrombin was administered at doses of 0, 12.5, 25, and 50 µg/kg and animals were evaluated for 4 days (main study) and 15 days (recovery study) after dosing. E-WE thrombin was well tolerated in both species and no E-WE thrombin-related AEs, apart from the temporal aPTT prolongation outside the reference range (the anticipated systemic effect at high doses), were observed. Based on these results, the no observed adverse effect level (NOAEL) was determined to be 300 µg/kg/dose and 50 µg/kg/dose, for rats and cynomolgus monkeys, respectively.

8.2.2 Clinical Studies

A first-in-human (FIH) study (Study No. EWE-17-01 [Celerion Project No.: CA19169]) was completed in the US on 25 November 2018. This study was a single ascending dose study in

healthy adult male and female subjects with the purpose of collecting safety, tolerability, and PD information in order to support further development of E-WE thrombin. A total of 21 male and female subjects were evaluated following IV bolus injection of one of four doses of E-WE thrombin (0.5, 1, 2, 4 µg/kg).

All 21 subjects who entered the study received the study medication or placebo and completed the study per protocol. Below is a summary of the preliminary data from this FIH study as of 24 of January 2019.

8.2.2.1 Safety Data

There were no deaths or serious AEs (SAEs) experienced in Study No. EWE-17-01 [Celerion Project No.: CA19169]. No subjects were discontinued due to AEs.

Overall, 4 of 21 (19%) subjects experienced a total of 8 treatment-emergent AEs (TEAEs) in this study, with 3 of 16 (19%) subjects following active treatment and 1 of 5 subjects (20%) following placebo. The majority of the events (5) were mild (Grade 1) in severity and 3 were moderate (Grade 2). Of the 8 TEAEs reported, only one was suspected to be possibly treatment related by the Principal Investigator (PI).

The most common TEAE was headache, experienced by 2 (13%) active-treatment subjects. One case of headache of moderate severity (Grade 2) was deemed to be possibly treatment related by the PI and had a reported onset of approximately 10 minutes after dosing, resolving 36 hours later. The other case of headache (Grade 1) was deemed to be unrelated to treatment by the PI and was reported 17 days after dosing, resolving 24 hours later. Other TEAEs reported included back pain, pruritus, papular rash and skin exfoliation in one placebo subject and vessel puncture site hemorrhage at the site of IV attempt in one active treatment subject.

Temporal aPTT prolongation was not evaluated as an AE, because it was an expected response, indicating a natural response to activation of endogenous protein C and release of some APC into systemic circulation.

A single IV dose of E-WE thrombin up to 4 µg/kg appeared to be safe and generally well tolerated by the healthy male and female subjects in this study.

8.2.2.2 Pharmacodynamic Data

The APC-PCI was used as a surrogate biomarker for drug exposure.

Mean plasma APC-PCI concentration results following single IV doses of E-WE thrombin are presented in Figure 1. Plasma APC-PCI was detectable in all subjects at the first sampling time point of 0.08 hour (5 minutes) post-injection and remained detectable throughout the entire sampling interval in all subjects up to 2 hours in the 0.5 µg/kg dose level group and up to 4 hours in the 1, 2 and 4 µg/kg dose level groups. Plasma concentrations of APC-PCI increased in a temporal and dose-dependent manner. All concentrations returned to baseline by 24 hours. Peak mean APC-PCI concentrations occurred at 0.5 hour (30 minutes) in all dose level groups. Ascending doses of E-WE thrombin increased the peak and overall

APC-PCI concentrations in a dose-proportional manner from the 0.5 to 2 µg/kg dose levels, and slightly less than dose-proportional from the 2 to 4 µg/kg.

Figure 1. APC-PCI Complexes after E-WE Thrombin Administration in Healthy Subjects

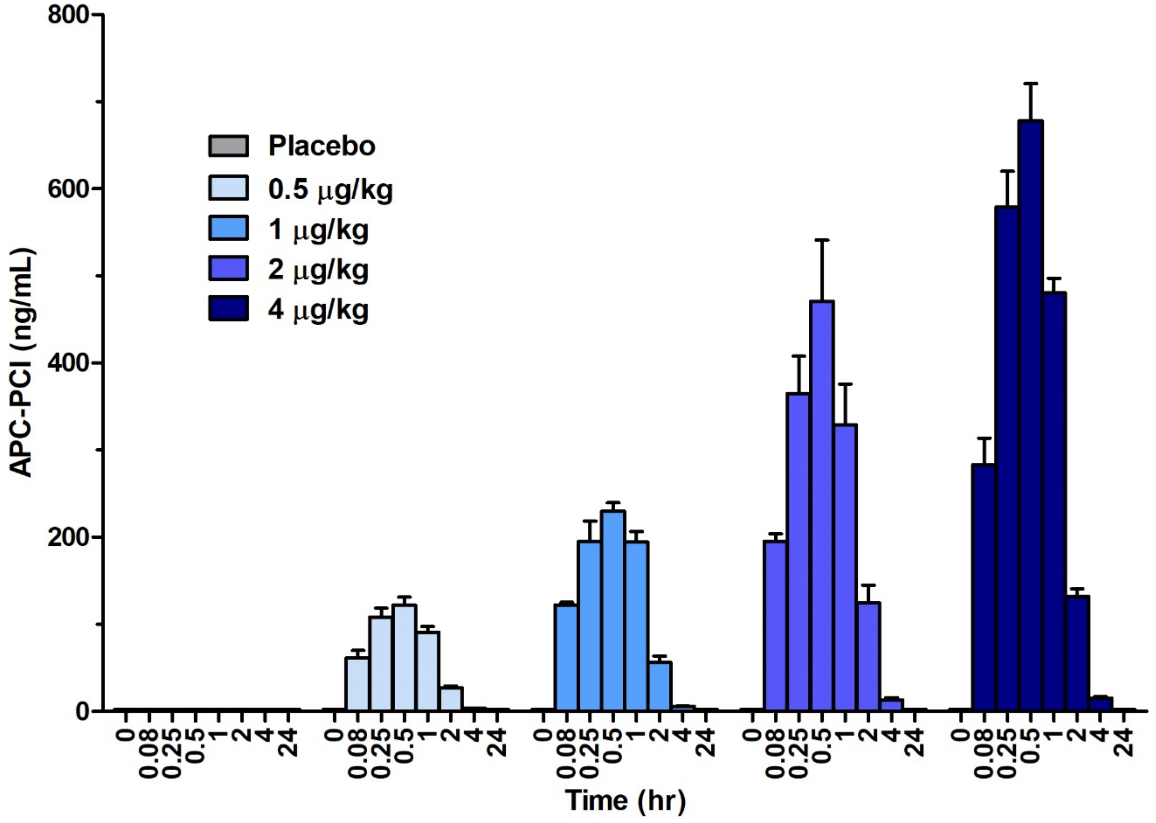

Anticoagulation was verified by a temporal, dose-dependent increase in the aPTT, which returned to baseline by 4 hours (in the 0.5 and 1 µg/kg groups) and by 24 hours (in the 2 and 4 µg/kg groups). Administration of E-WE thrombin did not significantly impact prothrombin time (PT), platelets or fibrinogen levels (Figure 2). In the figure, laboratory reference ranges are shown with black dashed lines and clinically significant ranges are shown with red dashed lines.

Figure 2. Coagulation Parameters after E-WE Thrombin Administration in Healthy Subjects

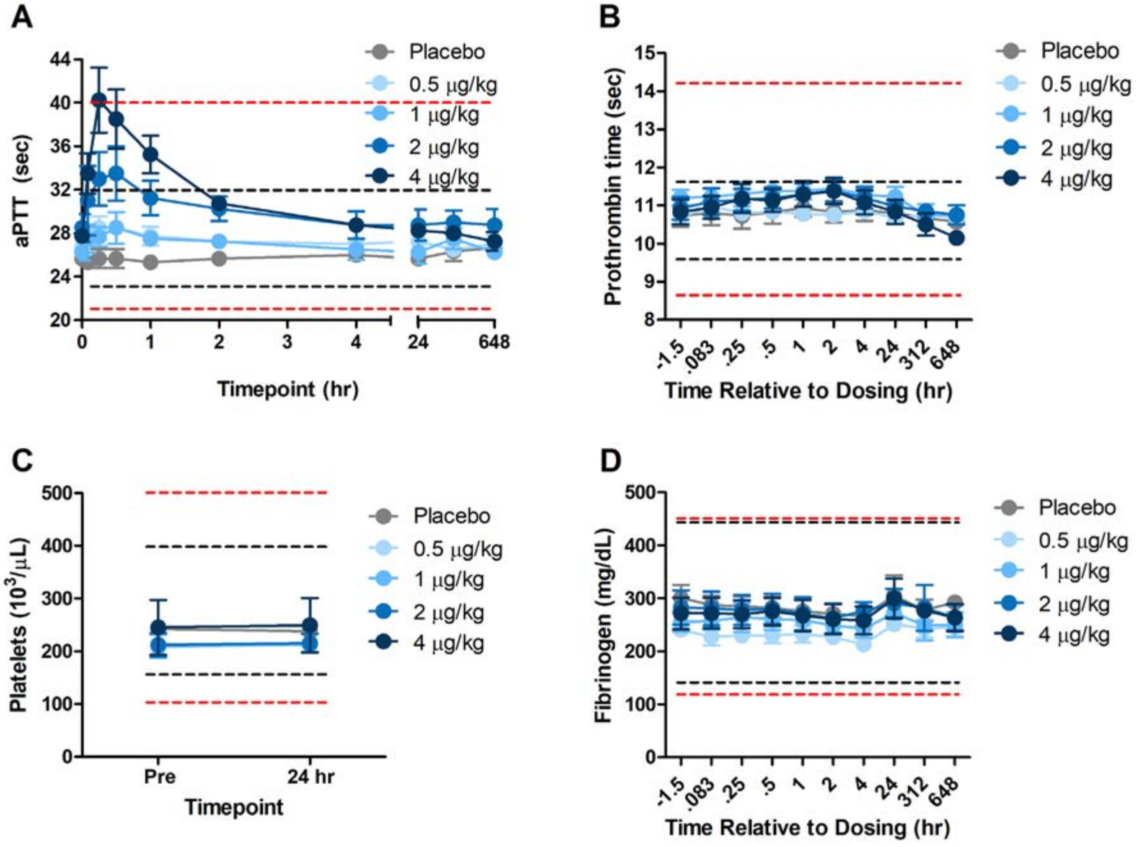

8.3 Rationale

8.3.1 Purpose of the Study Design

A FIH study was conducted in healthy subjects and concluded that a single dose of E-WE thrombin up to 4 µg/kg, administered as a bolus, was safe and generally well tolerated. This study will explore antithrombotic potential and utility of single doses of E-WE thrombin administered as a bolus followed by an infusion in the treatment and prevention of thrombus formation in an extracorporeal device such as an HD cartridge.

Thromboembolic disease and bleeding are common comorbidities in patients with chronic kidney diseases [5]. In most ESRD patients, heparin is routinely used during outpatient HD to reduce clotting in the dialyzer cartridge and to improve HD efficiency. However, administration of heparin during HD temporally increases the risk of bleeding. A safe alternative for patients with active bleeding, patients with previously documented or acquired heparin-induced thrombocytopenia, or that are at high risk for bleeding complications, is heparin-free dialysis [6-8] and the majority of HD patients that are admitted to the hospital are routinely dialyzed without heparin due to bleeding concerns.

In this study design, patients will be confined to the clinic for the majority of the trial and will not receive heparin during their HD sessions. While heparin-free HD can increase clotting within the dialyzer, resulting in the need for exchange of the dialyzer cartridge during HD in 5-7% of the procedures [9], there is no evidence that HD without heparin results in increased thromboembolic complications. Indeed, heparin anticoagulation during HD is not intended for systemic thromboprophylaxis and a recent retrospective analysis of data from the United States Renal Data System and electronic medical records shows that heparin-free HD is not associated with increased risk of thromboembolic events in ESRD patients [10]. Therefore, the removal of standard of care heparin anticoagulation in either arm for the short duration of the study is not expected to be a clinically relevant safety risk, nor place the patients at an increased risk for development of thromboembolic complications. Additionally, it enables the most rigorous assessment of safety and efficacy for E-WE thrombin that would not be possible if heparin anticoagulation during HD was maintained.

E-WE thrombin is expected to reduce thrombogenesis by downregulating thrombin generation in the extracorporeal circuit and hemodialyzer without clinically significant impairment of the patient's hemostasis or promoting thrombosis. Both thromboembolism and bleeding are common comorbidities in ESRD patients on HD, and heparin for HD is not well tolerated in some patients, as about 4% of ESRD patients develop heparin-induced thrombocytopenia (HIT). Severe HIT is a life-threatening acute complication of heparin exposure. HIT antibodies have been detected in about 8% of HD patients who receive unfractionated heparin, and some of them are at risk of developing a severe HIT event. Thus, an intrinsically safe short-term and self-limiting anticoagulant as an alternative to heparin is currently an unmet need for this patient population. As such, E-WE thrombin administration during HD may be an effective method to protect the hemodialyzer from clot development, and stands as a promising, short-acting alternative to other anticoagulants in this population. It is therefore of interest to assess E-WE thrombin safety and effectiveness in ESRD patients on chronic HD.

A placebo arm is included in this study design in order to robustly demonstrate the safety and efficacy of E-WE thrombin while not placing the patients at unnecessary risk, as mentioned above. The primary endpoint in this study is safety, measured as the number or frequency/rate of AEs that occur during the comparable observation periods. A heparin-free inpatient HD observation period (from Day -8 to Day 1 prior to dosing) is included in the trial design to establish a baseline for the heparin-free procedure. Continuation of the heparin-free HD procedure during the subsequent trial period and inclusion of a placebo arm allows for the determination of whether an AE is drug-related or not and permits accurate assessment of the safety of the study drug. The addition of a placebo arm also removes potential bias from the interpretation of AEs by the PIs and from the reporting by the patients. The secondary endpoint for this study is efficacy of E-WE thrombin on HD which may be more clearly observed with a placebo control instead of an active control (e.g., heparin). While the placebo arm may be at the same risk of clotting within the dialysis circuit as during the pre-dosing period (Days -8 to 1), data cited above suggests that these patients will not be at higher risk for either thromboembolic or bleeding complications. Should clotting occur within the dialyzer cartridge during a HD session that results in dialyzer failure, the dialyzer cartridge will be replaced, and the patient will be closely monitored by

the PI. It is important to note that replacement of the dialyzer cartridge during HD is safe for the patient and a routine procedure in cases of dialyzer failure.

8.3.2 Dose Selection

A FIH study was conducted in healthy subjects and concluded that a single bolus dose of E-WE thrombin up to 4 µg/kg was safe and generally well tolerated. Thus the 1.5 and 3 µg/kg doses were selected for this phase 2 study in order to provide adequate PD and efficacy characterization but still allowing a safety margin should the results differ in this population compared to healthy subjects. E-WE thrombin will be administered as a combination of a small bolus loading dose to induce rapid response but without significant systemic anticoagulation, and an infusion over 4 hours to prolong and sustain the duration of effect to cover the entire HD procedure.

8.3.3 Rationale for Pharmacodynamic Markers

The presumed half-life of active circulating E-WE thrombin in solution is very short (seconds to minutes) due to rapid binding to cell surface thrombin receptors. The active enzyme is not measurable in plasma using our enzyme capture assay at predicted human therapeutic doses. The Sponsor will use a PD biomarker (circulating APC-PCI complexes) as a surrogate for drug exposure.

8.4 Risk/Benefit

ESRD patients are often ill and are prone to a variety of AEs on or off HD, ranging, for example, from infections to hypertension to bleeding to thromboembolism to acute ischemic cardiovascular emergencies, even if they are apparently stable on chronic HD.

E-WE thrombin is a purified protein produced by recombinant DNA technology in a bacterial (Escherichia coli) cell culture system. All drugs, including heparins or recombinant proteins can occasionally and unpredictably evoke acute or chronic allergic reactions. Some ESRD patients develop chronic HD- and uremia-related moderate hemorrhagic diathesis or hemostasis impairment. The hemostatic safety of E-WE thrombin in the complex conditions of ESRD and chronic HD is not yet known. The safety monitoring practices employed by this protocol (i.e., AE questioning [including HD vascular access site reaction], physical examinations, bleeding time from the HD vascular access sites, vital sign measurements, 12-lead ECGs, clinical laboratory and coagulation tests) are adequate to protect the patients' safety and should detect all expected and unexpected TEAEs. In addition, the cohorts will be dosed in a sequential manner, starting with the lower dose level, Cohort 1.

There may be a higher risk of clots developing in the hemodialyzer during heparin-free HD. Should this occur, the site standard operating procedures will be followed to replace the clotted dialyzer and resume HD using a new cartridge under the supervision of the PI. It is important to note that replacement of the dialyzer cartridge is a safe procedure for the patient. While studies suggest that there is no risk of thromboembolic complications should this occur, complete clotting of the dialyzer circuit could result in up to ~228 mL of blood loss, and is usually prevented by replacing the dialyzer before complete occlusion.

As with all injectable recombinant or natural biologic products, subjects who receive these drug products may become immunized against the drug product. Subjects receiving E-WE thrombin may thus develop anti-drug antibodies (ADA), which may or may not neutralize the drug, or cross-react with and inhibit endogenous thrombin functions. While development of immunity and appearance of ADA in the circulation is not an AE as long as the ADA does not cross react with endogenous thrombin, the appearance of such antibodies will be monitored, as it may affect future tests and uses of the product, including future evaluation of repeat drug administration. Development of ADA that cross-react with the host's prothrombin/thrombin is a remote but existing possibility. All subjects will be assessed for the presence of antibodies to either E-WE thrombin or wildtype thrombin using validated assays.

There may be direct health benefits for study participants from receipt of study drug, some evaluated among the secondary outcome measures. E-WE thrombin has shown antithrombotic effects in nonclinical studies. Reduced thrombus accumulation on the dialyzer surface may potentially improve the efficiency of HD as well. An indirect health benefit for study participants enrolled in this study is the free medical tests received at screening and during the study.

9. STUDY OBJECTIVES AND ENDPOINTS

9.1 Study Objectives

The primary objective of this study is:

- To assess the safety and tolerability of a single dose of E-WE thrombin infused into the dialysis line during a 4-hour HD procedure in patients with ESRD.

The secondary objectives of this study are:

- To assess the PD of a single dose of E-WE thrombin infused into the dialysis line during a 4-hour HD procedure in patients with ESRD. APC-PCI will be used as a surrogate biomarker for drug exposure.

- To assess the efficacy of a single dose of E-WE thrombin infused into the dialysis line during a 4-hour HD procedure in patients with ESRD. Efficacy will be assessed by thrombus accumulation within the dialyzer cartridge (evaluated by visual inspection) and measurement of urea removed by 4 hours of dialysis (Kt/V and URR).

9.2 Study Endpoints

Primary outcome measures:

- The safety and tolerability of E-WE thrombin following active treatment will be evaluated versus pre-treatment and placebo by number and severity of AEs (including HD vascular access site reactions), physical examinations, bleeding time from the HD vascular access sites, vital sign measurements, 12-lead ECGs, clinical laboratory tests, and coagulation tests.

Secondary outcome measures:

- PD of E-WE thrombin will be measured by APC-PCI and plasma APC-PCI will act as a surrogate marker for E-WE thrombin drug exposure.

- Efficacy of antithrombotic activity will be evaluated by thrombus accumulation within the dialyzer cartridge (evaluated by visual inspection) after E-WE thrombin active treatment versus pre-treatment and placebo.

- Efficiency of HD will be assessed using BUN levels before and after dialysis to determine urea removed by 4 hours of dialysis (Kt/V and URR) after E-WE thrombin active treatment versus pre-treatment and placebo.

Other outcome measures:

- Development of antibodies to E-WE thrombin or WT thrombin after drug exposure.

10. INVESTIGATIONAL PLAN

10.1 Overall Study Design and Plan

This is a phase 2, randomized, double-blind, placebo-controlled, single-dose study of E-WE thrombin designed to evaluate the safety and efficacy at 2 dose levels administered to patients with ESRD during HD conducted at one study center in the US.

Patients will be enrolled in Cohort 1 or Cohort 2; cohorts will be dosed sequentially. Dosing of Cohort 2 will commence following completion of all study events for all patients in Cohort 1. Within each cohort, patients will be randomized to receive active drug or matching placebo as summarized below:

Table 1. Cohort and Treatment Randomization

Cohort	Treatment	Active:Placebo	Cohort Size
1	0.5 µg/kg bolus dose administered into the dialysis line immediately followed by a 0.25 µg/kg/hour continuous infusion dose into the dialysis line over 4 hours of E-WE thrombin or matching placebo, for a total dose of 1.5 µg/kg	2:1	12:6
2	1 µg/kg bolus dose administered into the dialysis line immediately followed by a 0.5 µg/kg/hour continuous infusion dose into the dialysis line over 4 hours of E-WE thrombin or matching placebo, for a total dose of 3 µg/kg	2:1	12:6

This study includes a screening period of 28 days prior to checking in to the CRU on Day -8. From Day -8 (8 days prior to dosing on Day 1) through Day 3, all patients will undergo HD 5 times and will be assessed for all scheduled procedures and endpoints before and after each HD session as per the Study Events Flow Chart (Section 6), using only one type of dialyzer cartridge for each patient.

On Day 1, prior to dosing, patients will undergo baseline measurements followed by initiation of a regular HD procedure, consistently using only one type of dialyzer cartridge for each patient. A single dose of E-WE thrombin or matching placebo will be administered into the dialysis line during the HD procedure. Patients will continue to undergo HD procedures and scheduled assessments from Day 1 through Day 3.

Study assessments will include AEs, including HD vascular access site (AV fistula or AV graft) reactions, physical examinations, vital sign measurements, 12-lead ECGs, clinical laboratory tests, coagulation tests, immunogenicity assessments, and PD blood sampling for APC-PCI and measurement of urea removed by 4 hours of dialysis to be performed throughout the study according to the Study Events Flow Chart (Section 6). On Days -7, -5, -2, 1, and 3, bleeding time from the HD vascular access sites on the AV fistula or AV graft (ladder or buttonhole technique) will be evaluated immediately at the end of the HD session. The dialyzer cartridge will be rinsed, visually inspected and graded against a

standardized visual assessment scale at the end of the HD procedure, and photographed on 4 sides.

Additionally, plasma and dialysate samples will be collected and sent to Sponsor along with the used and rinsed dialyzer cartridges for potential internal evaluation of plasma protein C, dialysate urea, and total protein accumulation within the dialyzer cartridge.

10.1.1 Confinement, Return Visits, and Follow-Up

Patients will be admitted to the CRU on Day -8, at the time indicated by the CRU, until after completion of study procedures on Day 3 as indicated in the Study Events Flow Chart (Section 6). At all times, a patient may be required to remain at the CRU for longer at the discretion of the PI or designee.

All patients who receive the study drug or matching placebo (including patients who terminate the study early) will return to the CRU on Day 14 (± 2 days) for coagulation tests, immunogenicity sample collection, and to determine if any AE has occurred since the last study visit.

10.1.2 Study Duration

The total planned duration of patient participation is approximately 50 days from screening to follow-up.

10.2 Study Conduct

See the Study Events Flow Chart for a summary of the schedule of study participation and procedures in Section 6. The end of the study is defined as the date of the last scheduled study procedure as outlined in the Study Events Flow Chart (Section 6).

11. STUDY POPULATION

11.1 Inclusion Criteria

Patients must fulfill all of the following inclusion criteria to be eligible for participation in the study:

1. ESRD maintained on stable outpatient HD regimen, using an established (> 3 months) and normally functioning, regular flow, uninfected mature AV fistula (or AV graft) and skin consistent with standard chronic HD access injuries, and HD stability defined as Kt/V ≥ 1.2 within 3 months prior to screening at a healthcare center for > 3 months from screening.

2. On HD regimen at least 3 times per week for a minimum of 3 hours per dialysis session, using a complication-free well maintained AV fistula (or AV graft), expected and plan to continue this throughout and for at least 3 months beyond the study.

3. Is capable of understanding the written informed consent, provides signed and witnessed written informed consent and agrees to comply with protocol requirements and study-related procedures.

4. Willing to be confined to the CRU for the duration of the study, able to comply with all study-related requirements, and able to adhere to study restrictions and visit schedules.

5. Male or female, between 18 and 80 years of age (inclusive) at the time of screening.

6. BMI of ≥ 18 at the time of screening.

7. Considered by the PI to be clinically stable with respect to underlying ESRD, based on the medical evaluation that includes medical and surgical history, and a complete physical examination including vital sign measurements, ECGs, and clinical laboratory and coagulation test results at screening. Repeat assessments are permitted for any laboratory, coagulation, ECG, or vital sign parameter required for enrollment.

8. Female patients must be of non-childbearing potential and must have undergone one of the following sterilization procedures at least 6 months prior to dosing:

    - hysteroscopic sterilization;
    - bilateral tubal ligation or bilateral salpingectomy;
    - hysterectomy;
    - bilateral oophorectomy;

    or be postmenopausal with amenorrhea for at least 1 year prior to dosing and follicle-stimulating hormone (FSH) serum levels consistent with postmenopausal status as per PI or designee judgment.

9. Male patients must either be sterile (vasectomy with history of a negative sperm count following the procedure); practice total abstinence from sexual intercourse as the preferred lifestyle (periodic abstinence is not acceptable); use a male condom with any sexual activity; or agree to use a birth control method considered to be appropriate by the Investigator from the time of dosing until 90 days after study drug administration. Male patients must agree not to donate sperm for a period of 90 days after study drug administration.

11.2 Exclusion Criteria

Patients must not be enrolled in the study if they meet any of the following criteria:

1. Documented history of acute vasoocclusive thrombotic event (acute coronary syndrome, stroke or transient ischemic attack, venous thromboembolic event), or vascular access fistula or AV graft failure in the past 3 months.

2. With the exception of unfractionated heparin during HD that is allowed until study check-in, concomitant or prior use of anticoagulant/antiplatelet agents (e.g., low molecular weight heparins, warfarin, apixaban, bivalirudin, ticagrelor, edoxaban, dabigatran, rivaroxaban, clopidogrel, prasugrel, ticlopidine, eptifibatide, tirofiban, dipyridamole, diclofenac, and all other non steroidal antiinflammatory drugs) that may affect hemostasis for 2 weeks prior to check-in on Day -8 and throughout the study.

3. Any clinically significant (CS) concomitant disease or condition (including treatment for such conditions) that, in the opinion of the PI, could either interfere with the study drug, compromise interpretation of study data, or pose an unacceptable risk to the patient.

4. Any other CS abnormalities in laboratory test results at screening or Day -8 check-in that would, in the opinion of the PI, increase the patient's risk of participation, jeopardize complete participation in the study, or compromise interpretation of study data.

5. Pregnant (positive pregnancy test) at screening or check-in on Day -8. If serum human chorionic gonadotropin (hCG) pregnancy test results are indeterminate, follow-up testing should be performed to determine eligibility.

   All female patients will not be pregnant and will have a negative pregnancy test at screening and check-in on Day -8, with the following exception: females receiving dialysis with an indeterminate pregnancy test result or persistently low hCG resulting in a false positive pregnancy test may be included in the study at the discretion of the PI. Postmenopausal patients with a result outside the postmenopausal range or an indeterminate pregnancy test will undergo additional testing with FSH to confirm postmenopausal status prior to study enrollment.

6. Treatment with another investigational drug or participation in a device study within 30 days (or 5 half-lives, whichever is longer) prior to check-in on Day -8.

7. Acute illness that is considered by the PI to be CS within 2 weeks of check-in on Day -8.

8. Surgery within the past 90 days prior to dosing which in the opinion of the PI or designee is clinically relevant.

9. Currently have established underlying inherited or acquired symptomatic bleeding disorders and/or are at risk for excessive bleeding per PI judgment or current active bleeding (e.g., gastrointestinal, intracranial), aside from minor bleeding from the puncture site on the AV fistula or AV graft, which would be expected to occur during the dialysis procedure, with the following values:
   - Platelet count < 100,000 cells/mm^3 (if < 100,000 cells/mm^3 but > 75,000 cells/mm^3, with permission of PI and medical monitor) at screening or check-in on Day -8
   - INR > 1.4 at screening or check-in on Day -8
   - aPTT up to 1.2 x upper limit of normal (ULN) (if > 1.2 x ULN and up to < 1.5 x ULN, with permission of PI and medical monitor) at screening or check-in on Day -8
   - Alanine aminotransferase (ALT) or aspartate aminotransferase (AST) > 2 x ULN at screening or check-in on Day -8
   - Total bilirubin > 1.2 x ULN at screening or check-in on Day -8

10. Seated blood pressure < 90/40 mmHg at screening and check-in on Day -8.

11. Exclusion criteria for ECG at screening and check-in on Day -8:

Heart rate	< 45 and > 110 bpm
QTcF interval	> 500 msec
bpm = beats per minute; msec = milliseconds; QTcF = QT interval corrected using Fridericia's formula	

- Any significant arrhythmia or conduction abnormality, (including but not specific to atrioventricular block [2nd degree or higher], Wolff Parkinson White syndrome [unless curative radio ablation therapy]), which, in the opinion of the PI and Medical Monitor, could interfere with the safety for the individual patient.
- Non-sustained or sustained ventricular tachycardia (> 2 consecutive ventricular ectopic beats at a rate of > 1.7/second).

12. History of a CS allergy or a known sensitivity or idiosyncratic reaction to any compound known to be present in E-WE thrombin, its related compounds, or any compound listed as being present in the study formulation.

13. Hypersensitivity to ß-lactam / penicillin derivatives.

14. Participate in strenuous exercise from 72 hours prior to check-in on Day -8 and throughout the study.

15. Positive test for drugs of abuse and/or positive alcohol test at screening or check-in on Day -8 if not accounted for by a prescription medication. Patients with a positive test based on a prescribed medication may be enrolled.

16. Positive test at screening for hepatitis B surface antigen (HBsAg) or human immunodeficiency virus (HIV). If a patient with ESRD has positive test results for hepatitis C virus (HCV) but liver function tests are otherwise not clinically significant, the patient may be included at the PI's discretion.

17. Receiving blood purification therapy other than HD.

18. Donation of blood or significant blood loss within 56 days prior to dosing.

19. Plasma donation within 7 days prior to dosing.

20. Presence of advanced malignant neoplasms of any organ or system that produces illness or symptoms that have been treated within 3 months with chemotherapy or whole body irradiation, or bone marrow irradiation, and may affect life expectancy in the following 6 months.

21. Any other reason that would render the patient unsuitable for study enrollment at the discretion of the PI.

11.3 Early Termination of Patients from the Study

Patient participation in this study may be discontinued for any of the following reasons:

1. Occurrence of any medical condition or circumstance that exposes the patient to substantial risk and/or does not allow the patient to adhere to the requirements of the protocol.

2. If the patient clots the hemodialyzer or the dialysis circuit in the pre-treatment observation period (from Day -8 to Day 1 prior to dosing) at least twice during one HD session, they may be discontinued from the study at the discretion of the PI. If the patient repeatedly clots the hemodialyzer or dialysis circuit during each HD session, continuation of the patient in the study will be at the PI's discretion. However, available data obtained from such subjects will be included in the analyses.

3. Any SAE, clinically significant AE, severe laboratory abnormality, intercurrent illness, or other medical condition that indicates to the PI that continued participation is not in the best interest of the patient.

4. Patient's decision to withdraw.

5. Requirement of prohibited concomitant medication.

6. Patient failure to comply with protocol requirements or study-related procedures.

7. Termination of the study by the Sponsor, Food and Drug Administration (FDA), Celerion, CRU, or other regulatory authorities.

8. In the case of incomplete dosing (e.g., a large droplet of study medication on the surface of the skin) as deemed by the PI (or designee) and/or Sponsor.

The clinical report will include reason(s) for patient withdrawals as well as details relevant to the patient withdrawal.

If a patient is withdrawn from the trial prior to study completion, the patient will undergo all procedures scheduled for early termination as the situation allows (see the Study Events Flow Chart (Section 6). Any patient withdrawn due to an AE (whether serious or non-serious) or clinically significant abnormal laboratory test values will be evaluated by the PI or a monitoring physician and will be treated and/or followed up until the symptoms or values return to normal or acceptable levels, as judged by the PI or designee.

11.4 Study Restrictions

11.4.1 Prohibitions and Concomitant Medication

Consumption of foods and beverages containing the following substances will be prohibited as indicated:

- Xanthines/caffeine: 48 hours prior to dosing and throughout the period of sample collection (small amounts of caffeine derived from normal foodstuffs e.g., 250 mL/8 oz./1 cup decaffeinated coffee or other decaffeinated beverage, per day, with the exception of espresso; 45 g/1.5 oz. chocolate bar, per day, would not be considered a deviation to this restriction);
- Alcohol: 48 hours prior to dosing and throughout the period of sample collection;

Concomitant medications will be listed, recorded, and allowed except for those listed as prohibited in the exclusion criteria in Section 11.2. It is understood and will be considered that treatment with certain drugs or cessation of treatment with certain drugs may have adverse effects and could interfere with the evaluation of the primary outcome measures of the study. Apart from anticoagulant/antiplatelet agents, no patient will be requested to stop taking any medication to be able to participate in the trial.

Acetaminophen (up to 2 g per 24 hours) or opioids may be administered, for fever or chronic pain control, at the discretion of the PI or designee. Administration of opioids for certain types of pain management may be allowed.

If deviations occur, the PI or designee in consultation with the Sponsor if needed will decide on a case-by-case basis whether the patient may continue participation in the study.

11.4.2 Meals

At the CRU, meals and/or snacks and fluids will be provided at appropriate times following check-in and throughout the study as per the clinical site standard procedure.

11.4.3 Activity

On Day 1, as dosing will be upon initiation of the HD session, patients will be dosed in the same position as required for their HD session. Patients will maintain that position for the whole HD session, as per standard HD procedure, except when they are supine or semi-reclined for other study procedures. At other times, there will be no specific activity restrictions. However, should AEs occur at any time, patients may be placed in an appropriate position.

Patients will be instructed to refrain from strenuous exercise which could cause muscle aches or injury, including contact sports at any time from 72 hours prior to check-in on Day -8 and throughout the study.

12. TREATMENTS

12.1 Treatments Administered

A separate pharmacy manual provided by the Sponsor will detail the preparation and treatment administration to be followed for this study.

E-WE thrombin will be supplied as a sterile, non-pyrogenic, preservative-free liquid for bolus and infusion.

E-WE thrombin will be supplied as 0.1 mg/mL, according to the planned dose (µg/kg) for each cohort, as noted below in Table 2.

Matching placebo will be supplied as sterile formulation buffer for bolus and infusion.

Table 2. Investigational Products

Product	Description
Test	E-WE thrombin (0.1 mg/mL)
Placebo	Matching placebo for E-WE thrombin (16.2 mM sodium citrate, 3.8 mM citric acid, 150 mM sodium chloride, 0.1% polysorbate 80, at pH 6)

An unblinded pharmacist will be responsible for providing E-WE thrombin or matching placebo to the blinded study personnel for administration as per the randomization scheme.

Patients will be enrolled in Cohort 1 or Cohort 2. Within each cohort, patients will be randomized to active drug or matching placebo as summarized in Table 1.

On Day 1, patients in each cohort will receive a single dose of E-WE thrombin or matching placebo as a bolus followed by an infusion into the dialysis line during a 4 hour HD procedure. Cohort 1 will be completed prior to initiation of Cohort 2.

Planned doses will be as follows:

Cohort 1: 0.5 µg/kg bolus dose administered into the dialysis line immediately followed by a 0.25 µg/kg/hour continuous infusion dose into the dialysis line over 4 hours of E-WE thrombin or matching placebo, for a total dose of 1.5 µg/kg.

Cohort 2: 1 µg/kg bolus dose administered into the dialysis line immediately followed by a 0.5 µg/kg/hour continuous infusion dose into the dialysis line over 4 hours of E-WE thrombin or matching placebo, for a total dose of 3 µg/kg.

Hour 0 will correspond to the beginning of E-WE thrombin bolus administration. The time of bolus administration and the times at which the infusion is started and stopped must be recorded.

The patient weight recorded at check-in will be used to calculate the study drug dose.

Should the need arise to change the infusion rate (i.e., due to an AE), changes to the infusion rate and the times at which those changes are made will be documented.

12.2 Dose Modification

The dose and administration of the study drug to any patient within a cohort may not be modified. If necessary, a patient must be discontinued for the reasons described in Section 11.3.

12.3 Method of Assigning Patients to Treatment Groups

Each patient will be assigned a unique identification number upon screening. Patients who complete the study screening assessments and meet all the eligibility criteria will be assigned a unique randomization identification number at the time of dosing, different from the screening number, and will receive the corresponding product, according to a randomization scheme generated at Celerion.

Patients will be enrolled in Cohort 1 or Cohort 2 with Cohort 1 to be completed prior to the initiation of Cohort 2.

In each cohort, an attempt will be made to enroll at least 50% of females and at least 30% of a race/ethnic minority group. An attempt will also be made to approximately match key characteristics, including sex, average age, and weight between cohorts.

In each cohort, patients will be randomized to receive either E-WE thrombin or matching placebo maintaining a 2:1 ratio

If replacement patients are used, the replacement patient number will be 100 more than the original (e.g., Patient No. 101 will replace Patient No. 1).

12.4 Blinding

This is a double-blind, placebo-controlled study.

12.4.1 Maintenance of Randomization

A computerized randomization scheme will be created by a Celerion statistician and it shall be considered blinded as per the following: the randomization is available only to the CRU pharmacy staff preparing the drug who will not be involved in any other aspect of the study including administration of the drug. It will not be made available to the Sponsor, patients, or members of the staff responsible for the monitoring and evaluation of safety assessments.

12.4.2 Procedures for Breaking the Blind Prior to Study Completion

One set of sealed envelopes containing the randomization code will be supplied to the PI or designee at the start of the study.

Breaking of the blind is expressly forbidden except in the event of a medical emergency where the identity of the drug must be known in order to properly treat the patient, or in the event of a safety interim analysis at the request of the Sponsor.

In the event of a medical emergency, it is requested that the PI or designee make every effort to contact the Study Monitor or designee prior to breaking the blind. If breaking the blind is required because of a medical emergency, the treatment identity would be revealed by the PI or designee, for that patient only. In the event that the emergency is one, in which it appears that the other patients may be at imminent risk, the blind may be broken for all patients dosed at that dose level. The unblinding will be properly documented in the study file.

In all cases where the code is broken, the PI or designee should record the date and reason for code breaking.

At the end of the study, envelopes will be retained or destroyed according to site procedures unless specified otherwise by the Sponsor.

12.4.2.1 Revealing of Randomization

In the absence of a medical emergency, the blinded randomization for this study will not be revealed until all data are entered in the database, edits checks are performed, queries closed, and the database is officially locked.

12.4.3 Treatment Compliance

A qualified designee (e.g. nurse) will be responsible for administering and monitoring the timed dose. Before and after the bolus/infusion dose, the qualified designee will visually inspect the syringe to ensure that the patient has received the entire dose. In the case of incomplete dosing, a few milliliters of blood could be drawn back into the dosing syringe and carefully reinjected to ensure administration of the complete dose; this will be reported.

13. STUDY ASSESSMENTS AND PROCEDURES

The Study Events Flow Chart (Section 6) summarizes the clinical procedures to be performed at each visit. Individual clinical procedures are described in detail below. Additional evaluations/testing may be deemed necessary by the PI or designee and/or the Sponsor for reasons related to patient safety.

For this study, the primary assessment is safety and tolerability. Safety will be determined by evaluating AEs (including HD vascular access site reactions), physical examinations, bleeding time from the HD vascular access sites, vital sign measurements, 12-lead ECGs, clinical laboratory parameters, including but not limited to, hematology, serum chemistry profile, coagulation, and urinalysis, as outlined in the Study Events Flow Chart (Section 6).

Any nonscheduled procedures required for urgent evaluation of safety concerns take precedence over all routine scheduled procedures.

13.1 Screening

Within 28 days prior to check-in on Day -8, medical history and demographic data, including name, sex, age, race, ethnic minority group, body weight (kg), height (cm), BMI (kg/m^2) and history of tobacco use will be reported. Each patient will have a physical examination, vital sign measurements (heart rate, blood pressure, temperature, and respiratory rate), 12-lead ECG, and the laboratory tests of hematological, coagulation, hepatic and renal function and additional tests as noted in Section 13.2.7.

13.2 Safety and Tolerability Assessments

13.2.1 Body Weight

Body weight (kg) will be measured daily up to Day 3 and before and after each HD session as outlined in the Study Events Flow Chart (Section 6).

13.2.2 Physical Examination

A full physical examination will be performed as per Study Events Flow Chart (Section 6) and will include, at a minimum, assessment of the following systems: skin, head, ears, eyes, nose and throat, respiratory system, cardiovascular system, gastrointestinal system, neurological condition, blood and lymphatic systems, and the musculoskeletal system.

Abbreviated physical examinations will include at the minimum, examination of respiratory, cardiovascular, and gastrointestinal systems, with the option for further examination of additional systems as necessary based on reported symptoms/AEs.

Symptom-driven physical examination may be performed at other times if deemed necessary by the PI or designee.

13.2.3 Vital Signs

Single measurements of body temperature, respiratory rate, blood pressure, and heart rate, will be measured as outlined in the Study Events Flow Chart (Section 6). Additional vital signs may be taken at any other times if deemed necessary.

Vital signs will be performed with patients in a seated position following a 5 minute rest period, except when they are supine or semi-reclined because of study procedures and/or AEs (e.g. nausea, dizziness) or if deemed necessary by the PI or designee.

When scheduled prior to HD, vital signs will be performed within 1 hour prior to the start of the HD. When scheduled post-dose, vital signs will be performed within approximately 15 minutes of the scheduled time point.

13.2.4 ECG Monitoring

Single 12-lead ECGs will be performed as outlined in the Study Events Flow Chart (Section 6). Additional ECGs may be taken at any other times if deemed necessary by the PI or designee.

ECGs will be performed with patients in a semi-recumbent position following a 5 minute rest period. All ECG tracings will be reviewed by the PI or designee.

When scheduled post-dose, ECGs will be performed within approximately 20 minutes of the scheduled time point.

13.2.5 Vascular Access Site Reaction Assessment

The monitoring of AEs will include assessment of HD vascular access fistula (or AV graft) site reactions. The preferred vascular access is an established AV fistula (or AV graft). Well established and reaction-free AV graft is an alternative, but less preferred access option, as it is more prone to complications. Patients who are hemodialyzed through catheters are not included in the study. Therefore, inspection of the AV fistula (or AV graft) and surrounding area will be performed. Any new abnormal findings inconsistent with past skin injuries as expected in patients on chronic HD will be reported as AEs. AV fistula (or AV graft) site reactions will be evaluated as outlined in the Study Events Flow Chart (Section 6). Prior to HD, the AV fistula (or AV graft) site will be examined for inflammation, infection, or flow abnormalities (including occlusion of the fistula or graft). When scheduled post-dose, injection site examinations will be performed within approximately 20 minutes after completion of the HD session. Reactions will be rated according to the FDA Guidance for Industry: Toxicity Grading Scale for Healthy Adult and Adolescent Volunteers Enrolled in Preventive Vaccine Clinical Trials [11] as follows:

Local Reaction	Mild (Grade 1)	Moderate (Grade 2)	Severe (Grade 3)
Pain	Does not interfere with activity	Repeated use of non-narcotic pain reliever > 24 hours or interferes with activity	Any use of narcotic pain reliever or prevents daily activity
Tenderness	Mild discomfort to touch	Discomfort with movement	Significant discomfort at rest
Erythema/Redness*	2.5 – 5 cm	5.1 – 10 cm	> 10 cm
Induration/Swelling**	2.5 – 5 cm and does not interfere with activity	5.1 – 10 cm or interferes with activity	> 10 cm or prevents daily activity
* In addition to grading the measured local reaction at the greatest single diameter, the measurement should be recorded as a continuous variable.<br>** Induration/swelling should be evaluated and graded using the functional scale as well as the actual measurement.			

13.2.6 Bleeding of Access Site

Evaluation of bleeding and bleeding time from the HD vascular access sites on the AV fistula or AV graft using the ladder or buttonhole technique will be performed as per Study Events Flow Chart (Section 6).

Following HD, the needle will be removed from the vascular access site and immediately followed by the placement of the dressing (i.e., gauze). Pressure will be applied manually by a clinical associate for 10 minutes before checking for evidence of bleeding around the dressing. If there is still some bleeding, pressure will be applied back at the site for 5 minutes. The site will then be checked again for evidence of bleeding; this procedure will be repeated every 5 minutes until bleeding has stopped. Once bleeding has stopped, a new gauze dressing is applied and then taped. To ensure consistency when applying the pressure, the clinical staff will have received adequate training and an attempt will be made to have the same associates perform this test.

The time to clot will be recorded.

13.2.7 Clinical Laboratory Tests

All tests listed below will be performed as per Study Events Flow Chart (Section 6). In addition, laboratory safety tests may be performed at various unscheduled time points, if deemed necessary by the PI or designee.

Hematology
- Hemoglobin
- Hematocrit
- Total and differential leukocyte count
- Red blood cell count
- Platelet count

Coagulation
- PT/INR
- aPTT
- Fibrinogen
- Thrombin time (TT)

Serum Chemistry*
- BUN
- Bilirubin (total and direct)
- Alkaline phosphatase
- AST
- ALT
- Lactate dehydrogenase
- Albumin
- Sodium
- Potassium
- Bicarbonate
- Chloride
- Glucose
- Creatinine**

Urinalysis†
- pH
- Specific gravity
- Protein***
- Glucose
- Ketones
- Bilirubin
- Blood***
- Nitrite***
- Urobilinogen
- Leukocyte esterase***

Additional Tests
- HIV test
- HBsAg
- HCV
- Saliva drug screen
  - ➢ Opiates
  - ➢ Amphetamines
  - ➢ Cocaine
  - ➢ Cannabinoids
- Saliva or breath alcohol screen
- Serum pregnancy test (for females only)
- FSH (for postmenopausal females only)

* Samples for serum chemistry will be obtained following a fast of at least 4 hours, however, in case of dropouts or rechecks, patients may not have fasted for 4 hours prior to the serum chemistry sample is taken.

** At screening, creatinine clearance will be calculated using the Cockcroft-Gault formula.

*** If urinalysis is positive for protein, blood, nitrite and/or leukocyte esterase, a microscopic examination (for red blood cells, white blood cells, bacteria, casts, and epithelial cells) will be performed.

† May not be performed for anuric patients.

13.2.8 Adverse Events

13.2.8.1 Adverse Event Definition

AE will be defined as any untoward medical occurrence during the study, associated or not with the use of E-WE thrombin in the study patients, whether or not considered drug-related. TEAE is an AE that occurs after administration of the study drug or matching placebo.

A suspected adverse reaction means any AE for which there is a reasonable possibility that the drug caused the AE. Reasonable possibility means there is evidence to suggest a causal relationship between the drug and the AE.

An AE can, therefore, be any unfavorable and unintended sign (including an abnormal laboratory finding), symptom, or disease that is temporally associated with the use of a medicinal (investigational) product, whether or not related to the medicinal (investigational) product [12].

13.2.8.2 Monitoring

Patients will be monitored for AEs from check-in through completion of the study. Patients will be asked how they are feeling once per day during confinement and at each return/follow-up visit.

AEs (whether serious or non-serious) and clinically significant abnormal laboratory test value(s) will be evaluated by the PI or designee and treated and/or followed up until the symptoms or value(s) return to normal, or acceptable levels, as judged by the PI or designee.

Treatment of SAEs will be performed by a licensed medical provider, either at the CRU or at a nearby hospital emergency room. Where appropriate, medical test(s) and/or examination(s) will be performed to document resolution of event(s). The outcome may be classified as resolved, improved, unchanged, worse, fatal, or unknown (lost to follow up).

13.2.8.3 Reporting

All AEs that occurred during this clinical study (i.e., before, during, and after exposure of ESRD patients to E-WE thrombin) will be recorded. This design is to improve Sponsor ability to obtain reliable initial information about drug safety yet reducing the number of study patients needed to generate useful information.

AEs and SAEs, including those related to study participation (e.g., protocol-mandated intervention), will be recorded from the time of patient check-in until study completion.

AEs will be coded using the most current Medical Dictionary for Regulatory Activities (MedDRA)® available at Celerion.

The PI or designee will review each event and assess its relationship to study drug (likely, probably, possibly, unlikely, or unrelated) and severity. The date and time of onset, time relationship to drug dosing, duration, and outcome of each event will be noted.

Relationship of AE:

The relationship of each AE to the study drug will be assessed using the following definitions:

Unrelated	▪ The adverse event must clearly be caused by the participant's clinical state, or the study procedure/conditions<br>▪ Definitely not related to drug<br>▪ Temporal sequence of an adverse event onset relative to administration of drug not reasonable<br>▪ Another obvious cause of an adverse event
Unlikely	▪ Time sequence is unreasonable<br>▪ There is another more likely cause for an adverse event
Possibly	▪ Corresponds to what is known about the drug<br>▪ Time sequence is reasonable<br>▪ Could have been due to another equally, likely cause
Probably	▪ Is a known effect of the drug<br>▪ Time sequence from taking drug is reasonable<br>▪ Ceases on stopping the drug<br>▪ Cannot be reasonably explained by the known characteristics of the participant's clinical state
Likely	▪ Is a known effect of the drug (e.g. listed in IB)<br>▪ Time sequence from administering drug is reasonable<br>▪ Event stops upon stopping drug and returns upon restarting drug

Severity of AE:

Severity rating used during the study will be based on the toxicity grading scale tables present in the FDA (Center for Biologics Evaluation and Research) toxicity grading scale for healthy volunteers 4-point severity scale (Grade 1, 2, 3 and 4) [11].

The following definitions for rating severity will be used for AEs not identified in the guidance:

Mild (Grade 1)	The AE does not interfere with daily activity.
Moderate (Grade 2)	The AE interferes with daily activity, but no medical intervention is required.
Severe (Grade 3)	The AE prevents daily activity and requires medical intervention.
Potentially Life-threatening (Grade 4)	Emergency room visit or hospitalization is required.

13.2.8.4 Serious Adverse Events

If any AEs are serious, as defined by the FDA Code of Federal Regulations (CFR), Chapter 21, special procedures will be followed. All SAEs will be reported to the Sponsor via fax or e-mail within one working day of becoming aware of the event, whether or not the serious events are deemed drug-related. All serious event reporting will adhere to 21 CFR 312.32 for Investigational New Drugs (IND) and to the Guidance for Industry and Investigators: Safety Reporting Requirements for INDs and BA/BE, dated December 2012 [12]. The institutional review board (IRB) will be notified of the Alert Reports as per FDA regulations.

An SAE is any AE or suspected adverse reaction that in the view of either the PI (or designee) or Sponsor, results in any of the following outcomes: Death, a life-threatening adverse event, inpatient hospitalization or prolongation of existing hospitalization, a persistent or significant incapacity or substantial disruption of the ability to conduct normal life functions, or a congenital anomaly/birth defect. Important medical events that may not result in death, be life-threatening, or require hospitalization may be considered serious when, based upon appropriate medical judgment, they may jeopardize the patient or subject and may require medical or surgical intervention to prevent one of the outcomes listed in the above definition. Examples of such medical events include allergic bronchospasm requiring intensive treatment in an emergency room or at home, blood dyscrasias or convulsions that do not result in inpatient hospitalization, or the development of drug dependency or drug abuse.

Life-threatening is defined as an AE or suspected adverse reaction that in the view of the PI (or designee) or Sponsor, its occurrence places the patient or subject at immediate risk of death. It does not include an AE or suspected adverse reaction that, had it occurred in a more severe form, might have caused death.

Unexpected is defined as an AE or suspected adverse reaction that is not listed in the IB or is not listed at the specificity or severity that has been observed; or is not consistent with the risk information described in the general investigational plan or elsewhere in the current application, as amended.

If a SAE occurs to a patient on this study, contact the Sponsor personnel listed in Section 3.

13.3 Hemodialysis Procedure and Blood Clots in the Dialyzer Cartridge

Patients will undergo HD 5 times from Day -7 to Day 3 as delineated in the Study Events Flow Chart (Section 6). For each patient, the HD will last approximately 4 hours as per patient standard schedule which will be the same throughout the study.

Clots may develop in the HD circuit when patients are undergoing heparin-free HD. Pressure within the HD circuit is continuously monitored, and an increase automatically signals development of blockage. Should clotting within the circuit occur, site standard operating procedures will be followed to replace the clotted dialyzer and resume HD. It may be performed under the supervision of the PI, if appropriate. It is important to note that replacement of the dialyzer cartridge is a safe procedure for the patient. While studies

suggest that there is no risk of thromboembolic complications should this occur, clotting and complete occlusion of the dialyzer circuit could result in up to ~228 mL of blood loss. This is preventable by timely intervention and replacement.

The hemoglobin levels will be followed more closely in patients that require replacement of the dialyzer during HD.

If the patient clots the hemodialyzer or the dialysis circuit in the pre-treatment observation period (from Day -8 to Day 1 prior to dosing) at least twice during one HD session, they may be discontinued from the study at the discretion of the PI. If the patient repeatedly clots the hemodialyzer or dialysis circuit during each HD session, continuation of the patient in the study will be at the PI's discretion. However, available data obtained from such subjects will be included in the analyses.

13.4 Immunogenicity

Blood samples for immunogenicity assessment will be performed as per Study Events Flow Chart (Section 6).

13.4.1 Blood Sampling and Processing for Immunogenicity

Blood samples for immunogenicity assessments (i.e., E-WE thrombin and WT thrombin) will be performed as per Study Events Flow Chart (Section 6).

For the determination of E-WE thrombin ADA, blood samples will be collected in 1 blood collection tube of 4.5 mL, containing 3.2% sodium citrate, and processed into three plasma aliquots of at least 0.5 mL each.

For the determination of antibodies to WT thrombin, whole blood samples will be collected in a 5 mL blood collection tube, without anticoagulant, and processed to three serum aliquots of at least 0.5 mL each.

Samples will be shipped to the analytical laboratory for analysis at the end of the study.

Instructions for blood sampling, collection, processing, and sample shipment will be provided separately.

13.4.2 Analytical Method

Assessment of ADA will be performed by Haemtech Biopharma Services, Inc. (refer to Section 3). Development of antibodies to E-WE thrombin or WT thrombin will be monitored using a multi-tiered approach that will include screening, confirmatory, and if applicable, antibody titer and neutralizing antibody assays.

13.5 Pharmacodynamic Assessment – APC-PCI

13.5.1 Blood Sampling and Processing

For all patients, blood samples for the determination of APC-PCI will be collected in 4 mL sodium heparin blood collection tubes at scheduled time points as delineated in the Study Events Flow Chart (Section 6) and processed to plasma.

The sample times and Hour 0 are in relation to the beginning of E-WE thrombin bolus administration.

The allowable time deviation window for sample collection is as follows:

Scheduled Post-dose Sampling Time	Allowed Time Deviation Window
0.0 - 4 hours	≤ ± 2 minutes
> 4 - 24 hours	≤ ± 10 minutes
> 24 hours	≤ ± 30 minutes

At the end of the study, platelet poor plasma samples obtained by centrifugation of heparinized blood will be shipped to the analytical laboratory for analysis.

Instructions for blood sampling, collection, processing, and sample shipment will be provided separately.

13.5.2 Analytical Method

Plasma sample analysis for APC-PCI will be performed by Celerion using a qualified enzyme-linked immunosorbent assay method.

13.6 Pharmacodynamic Assessments – Drug Efficacy and Efficiency of HD

Pharmacodynamic assessments will include evaluation of the efficacy of E-WE thrombin and the efficiency of HD. For all patients, PD assessments will be performed at scheduled time points as delineated in the Study Events Flow Chart (Section 6).

Efficacy of antithrombotic activity will be evaluated by thrombus accumulation:

- thrombus accumulation will be evaluated by visual inspection of the dialyzer membrane at the end of HD procedures and by a gradation using a standardized visual assessment scale [11]. The dialyzer cartridge will be rinsed, visually inspected, graded, and photographed on 4 sides. The number of dialyzer cartridges used for each HD session will be recorded.

Efficiency of HD will be assessed by using BUN levels:

- BUN levels before and after dialysis will be used to determine urea removed by 4 hours of dialysis (Kt/V and URR) after E-WE thrombin active treatment versus pre-treatment and placebo.

13.7 Internal Analyses

The following analyses may be performed by the Sponsor for internal purposes and results will not be included in the study report.

13.7.1 Protein C Analysis

For all patients, blood samples for the determination of protein C will be collected in 2 mL blood collection tubes containing 3.2% sodium citrate at scheduled time points as delineated in the Study Events Flow Chart (Section 6) and processed to plasma.

The sample times and Hour 0 are in relation to the time of E-WE thrombin bolus administration.

The allowable time deviation window for sample collection is as follows:

Scheduled Post-dose Sampling Time	Allowed Time Deviation Window
0.0 - 4 hours	≤ ± 2 minutes
> 4 - 24 hours	≤ ± 10 minutes
> 24 hours	≤ ± 30 minutes

Platelet poor plasma samples obtained by centrifugation of citrated blood will be shipped to Aronora.

Instructions for blood sampling, collection, processing, and sample shipment will be provided separately.

13.7.2 Dialysate Analysis

A 10 mL dialysate sample will be taken as delineated in the Study Events Flow Chart (Section 6).

Instructions for the sample collection, processing, and sample shipment will be provided separately.

The samples will be sent to Aronora and will be stored for up to 5 years following the last dosing for future analysis.

13.7.3 Analysis of Protein Accumulation Within the Dialyzer Cartridge

Following visual assessment and photography of the HD cartridge at the end of the procedure the used and rinsed dialyzer cartridge will be frozen and sent to Aronora for total protein accumulation evaluation.

Instructions for the cartridge collection, processing, photography and cartridge shipment will be provided separately.

13.8 Blood Volume for Study Assessments

Table 3. Blood Volume during the Study

Sample Type	Number of Time Points	Approximate Volume per Time Point * (mL)	Approximate Sample Volume Over Course of Study (mL)
Screening laboratory safety tests (including hematology, serum chemistry, serology, and coagulation), FSH (for postmenopausal female patients only) and serum pregnancy (for female patients only).	1	16	16
On-study hematology and serum chemistry (this includes serum pregnancy for female patients only when scheduled at the same time)	2	12.5	25
On-study coagulation (aPTT, PT/INR, fibrinogen, and TT)	12	3.5	42
On-study platelet count only **	Up to 10	3.5	Up to 35
On-study BUN only **	Up to 10	3.5	Up to 35
Blood for PD (APC-PCI) (ships to Celerion)	8	4	32
Blood for protein C (ships to Aronora)	16	2	32
Blood for plasma immunogenicity (E-WE thrombin plasma ADA) (ships to Haemtech Biopharma)	2	4.5	9
Blood for serum immunogenicity (WT thrombin serum ADA) (ships to Haemtech Biopharma)	2	5	10
		Total Blood Volume (mL)→	Up to 236***

* Represents the largest collection tube that may be used for this (a smaller tube may be used).

** Platelet count and BUN values will be taken from the hematology, serum chemistry samples when these are scheduled to be collected at the same time. Samples will not be taken twice at the same timepoint.

*** If additional safety or PD analysis is necessary to obtain sufficient plasma/serum for analysis, additional blood may be obtained (up to a maximum of 50 mL).

14. STATISTICAL CONSIDERATIONS

Data will be handled and processed according to Celerion Standard Operating Procedures, which are written based on the principles of GCP.

14.1 Sample Size Determination

The sample size chosen for this study was selected without statistical considerations. The sample size of 36 patients in total (18 per cohort with a ratio of 2:1 of active study drug:matching placebo) has been designed to meet the study safety, tolerability, and PD objectives.

14.2 Population for Analyses

Safety Population: All patients who received the study drug (active or matching placebo) will be included in the safety evaluations.

PD Population: All patients who received the study drug (active or matching placebo) and had at least one post-dose measurement of any of the PD assessment will be included in the PD evaluations.

14.3 Statistical Analyses

Detailed methodology for summary and statistical analyses of the data collected in this study will be documented in a Statistical Analysis Plan (SAP). The SAP will be prepared by Celerion and agreed upon with the Sponsor. This document may modify the plans outlined in the protocol; however, any major modifications of the primary endpoints definition and/or its analysis will also be reflected in a protocol amendment. Additional statistical analyses other than those described in this section may be performed if deemed appropriate.

14.3.1 Safety and Tolerability Analyses

All safety data will be populated in the individual CRFs. All safety data will be listed by patients.

The placebo patients from all cohorts will be pooled into a single placebo group for all summaries and presentations.

Dosing dates and times will be listed by patient.

Incidence and time of bleeding from the HD access sites on the AV fistula (or AV graft), as described in Section 13.2.6, will be counted and recorded. Active treatment versus pre-treatment, placebo, and post-treatment results will be compared.

AEs will be coded using the most current version of Medical Dictionary for Regulatory Activities (MedDRA®) available at Celerion and summarized by treatment (2 active dose levels and pooled placebo) for the number of patients reporting the treatment emergent adverse event (TEAE) and the number of TEAEs reported. A by-patient AE data listing including verbatim term, coded term, treatment, severity, and relationship to study drug will be provided.

Safety data including ECGs, vital signs assessments, clinical laboratory results, and vascular access site reaction will be summarized by treatment (2 active dose levels and pooled placebo) and point of time of collection.

Descriptive statistics using appropriate summary statistics will be calculated for quantitative safety data as well as for the difference to baseline, when appropriate. In addition, a shift table describing out of normal range shifts will be provided for clinical laboratory results.

Concomitant medications will be listed by patient and treatment (2 active dose levels and pooled placebo) and coded using the most current version of the World Health Organization drug dictionary available at Celerion. Medical history will be listed by patient.

14.3.2 Immunogenicity Analyses

When applicable, antibody titer will be reported and summarized descriptively.

14.3.3 Pharmacodynamic Analyses

Placebo patients from Cohort 1 and Cohort 2 will be pooled for analysis.

Individual, mean, and median data for APC-PCI will be presented graphically for all treatments (2 active dose levels and pooled placebo).

Analysis will include assessments of drug efficacy and efficiency of HD. These will include changes in thrombus accumulation and measurement of urea removed by 4 hours of dialysis (Kt/V and URR).

The change from baseline in endpoints will be summarized by treatment (2 active dose levels and pooled placebo) and at each scheduled time point using descriptive statistics. The baseline values are any value observed from Day -8 to Day 1, prior to E-WE thrombin administration. An average of predose values may be used as baseline, as applicable. Any values after a single dose of E-WE thrombin are regarded as post-baseline values.

The change from baseline variables will be calculated as the post-baseline value minus the value at baseline.

Additional analyses may be performed as deemed necessary upon review of the data.

15. STUDY ADMINISTRATION

15.1 Ethics

15.1.1 Institutional Review Board

This protocol will be reviewed by the IRB, and the study will not start until the IRB has approved the protocol or a modification thereof. The IRB is constituted and operates in accordance with the principles and requirements described in the US Code of Federal Regulations (21 CFR Part 56). The IRB is compliant to International Council on Harmonisation (ICH) guidelines.

15.1.2 Ethical Conduct of the Study

This research will be carried out in accordance with the protocol, US Code of Federal Regulations, 21 CFR Parts 50, 56, and 312, the ethical principles set forth in the Declaration of Helsinki, GCP, and the ICH harmonized tripartite guideline regarding GCP (E6[R2] Consolidated Guidance, November 2016).

15.1.3 Subject Information and Consent

The purpose of the study, the procedures to be carried out and the potential hazards will be described to the subjects in non-technical terms. Subjects will be required to read, sign and date an ICF summarizing the discussion at screening, and will be assured that they may withdraw from the study at any time without jeopardizing their medical care.

Subjects will be given a copy of their signed ICF.

15.1.4 Confidentiality

All members of the Investigator's staff have signed confidentiality agreements. By signing this protocol, the Investigator and investigational staff will regard all information provided by the Sponsor and all information obtained during the course of the study as confidential.

The Investigator must guarantee the privacy of the subjects taking part in the study. Subjects will be identified throughout documentation and evaluation by a unique subject study number. Throughout the study, a subject's source data will only be linked to the Sponsor's clinical study database or documentation via a unique identification number. If subject name appears on any study document, it must be redacted before the copy of the documents is supplied to the Sponsor. Any information concerning the subjects (clinical notes, identification numbers, etc.) must be kept on file by the Investigator who will ensure that it is revealed only to the Sponsor, IRB, or regulatory authorities for the purposes of trial monitoring, auditing or official inspections. As required, in the case of an event where medical expenses are the responsibility of the Sponsor, personal information, i.e., full name, social security details, etc., may be released to the Sponsor. Appropriate precautions will be taken to maintain confidentiality of medical records and personal information in strictest confidence and in accordance with local data protection laws.

15.2 Termination of the Study

Celerion/CRU reserves the right to terminate the study in the interest of patient welfare.

The Sponsor reserves the right to suspend or terminate the study at any time.

15.3 Data Quality Assurance

Standard operating procedures are available for all activities performed at Celerion relevant to the quality of this study. Designated personnel of Celerion will be responsible for implementing and maintaining quality assurance (QA) and quality control systems to ensure that the trial is conducted, and that data are generated, documented and reported in compliance with the study protocol, GCP and GLP requirements as well as applicable regulatory requirements and local laws, rules and regulations relating to the conduct of the clinical study.

The Clinical Study Report will be audited by the QA department and the QA audit certificate will be included in the study report.

All clinical data will undergo a 100% quality control check prior to clinical database lock. Edit checks are then performed for appropriate databases as a validation routine using SAS® to check for missing data, data inconsistencies, data ranges etc. Corrections are made prior to the statistical database lock.

15.4 Direct Access to Source Data/Documents

The PI must maintain, at all times, the primary records (i.e., source documents) of each patient's data. Examples of source documents are laboratory reports, drug inventory, study drug label records, and CRFs that are used as the source.

Celerion/CRU will ensure that the Sponsor, IRB, and inspectors from domestic and foreign regulatory authorities will have direct access to all study-related sites, source data/documents, and reports for the purpose of monitoring, auditing (ICH[E6(R2)] 5.1.2 & 6.10) and inspection. In the event that other study-related monitoring should be done by other parties, they will be required to sign a confidentiality agreement prior to any monitoring and auditing.

15.5 Drug Supplies, Packaging and Labeling

The Sponsor will supply sufficient quantities of E-WE thrombin and matching placebo to allow completion of this study. The lot numbers, manufacture dates, and expiration dates (if available) of the drugs supplied will be recorded in the final report.

The investigational product(s) will be shipped to a designee at the CRU and must be stored in a pharmacy or locked and secured in a storage facility with controlled temperature. The temperature and humidity in the room will be monitored. The room is accessible only to those individuals authorized by the PI.

Records will be made of the receipt and dispensing of the study drugs (E-WE thrombin and matching placebo) supplied. At the conclusion of the study, any unused study drugs

(including placebo) as well as original containers (even if empty), will be retained by the CRU, returned to the Sponsor or designee, or destroyed, as per Sponsor instructions. If no supplies remain, this fact will be documented in the pharmacy product accountability records.

15.6 Data Handling and Record Keeping

Celerion standard CRFs will be supplied. CRFs are produced, stored electronically, and are available to the designated study team members. Each CRF is reviewed and signed by the PI. The final signed CRFs are provided to the Sponsor on CD.

All raw data generated in connection with this study, together with the original copy of the final report, will be retained by the CRU until at least 5 years after the last approval of a marketing application in an ICH region and until there are no pending or contemplated marketing applications in an ICH region or at least 5 years have elapsed since the formal discontinuation of clinical development of the investigational product. These documents should be retained for a longer period, however, if required by the applicable regulatory requirements or by an agreement with the Sponsor. It is the responsibility of the Sponsor to inform the PI/Institution as to when these documents no longer need to be retained.

15.7 Report Format

According to the ICH Harmonized Tripartite Guideline (Organization of the Common Technical Document for the Registration of Pharmaceuticals for Human Use M4 and the ICH M2 Expert Working Group), the final report will be written according to the ICH E3 Guideline (Structure and Content of Clinical Study Reports).

15.8 Protocol Amendments

Any amendments to the study protocol that seem to be appropriate as the study progresses will be discussed between Sponsor and the PI or designee. All revisions and/or amendments to the protocol in writing must be approved by the Sponsor, the PI or designee, and the IRB before implementation.

15.9 Publication Policy

All unpublished information given to Celerion or the CRU by the Sponsor shall not be published or disclosed to a third party without the prior written consent of the Sponsor.

The data generated by this study are considered confidential information and the property of the Sponsor. This confidential information may be published only in collaboration with participating personnel from the Sponsor or upon Sponsor's written consent to publish the article.

16. REFERENCES

1. Aronora, Inc.: E-WE thrombin, AB002. Investigator's Brochure. Version Number 2; 25 February 2019.
2. Berny MA., et al., Thrombin mutant W215A/E217A acts as a platelet GPIb antagonist. Arterioscler Thromb Vasc Biol, 2008. 28(2): p. 329-34.
3. Berny-Lang MA., et al., Thrombin mutant W215A/E217A treatment improves neurological outcome and reduces cerebral infarct size in a mouse model of ischemic stroke. Stroke, 2011. 42(6): p. 1736-41.
4. Gruber, A., et al., Relative antithrombotic and antihemostatic effects of protein C activator versus low-molecular-weight heparin in primates. Blood, 2007. 109(9): p. 3733-40.
5. Lutz J., Jurk K., and Schinzel H. Direct oral anticoagulants in patients with chronic kidney disease: patient selection and special considerations. Int J Nephrol Renovasc Dis. 2018;10:135-143. doi: 10.2147/ijnrd.s105771
6. Schwab S.J., Onorato J.J., Sharar L.R., et al. Hemodialysis without anticoagulation. One-year prospective Trial in Hospitalized Patients at Risk for Bleeding. Am J Med. 1987;83:405-410.
7. Stamatiadis D.N., Helioti H., Mansour M., et al. Hemodialysis for patients bleeding or at risk for bleeding, can be simple, safe and efficient Clin Nephrol. 2004;62(1):29-34.
8. Caruana R. J., Raja M.R., and Bush J.V., et al. Heparin free dialysis: comparative data and results in high risk patients. Kidney Int. 1987;31:1351-1355.
9. Cronin R.E., and Reilly R.F. Unfractionated heparin for hemodialysis: still the best option. Semin Dial 2010;23(5):510-515. Doi: 10.1111/j.1525-139X.2010.00770.x.
10. Shen J.I., Mitani A.A., Chang T.I., et al. Use and safety of heparin-free maintenance hemodialysis in the USA. Nephrol Dial Transplant. 2013 Jun;28(6):1589-602. doi: 10.1093/ndt/gft067.
11. FDA Guidance for Industry: Toxicity Grading Scale for Healthy Adult and Adolescent Volunteers Enrolled in Preventive Vaccine Clinical Trials. September 2007. Available online: http://www.fda.gov/downloads/BiologicsBloodVaccines/GuidanceComplianceRegulatoryInformation/Guidances/Vaccines/ucm091977.pdf
12. FDA Guidance for Industry and Investigators: Safety Reporting Requirements for INDs and BA/BE, dated December 2012. Available online at: http://www.fda.gov/downloads/Drugs/.../Guidances/UCM227351.pdf
13. Ronco C., Brendolan A., Nalesso F., et al. Prospective, randomized, multicenter, controlled trial (TRIATHRON 1) on a new antithrombogenic hydrophilic dialysis membrane. Int J Artif Organs 2017;40(5):234-9
