# Supplementary material for: Safety and tolerability of the protein C activator AB002 in end-stage renal disease patients on hemodialysis: a randomized phase 2 trial
Source: Commun Med (Lond). 2024 Jul 26;4:153. doi: 10.1038/s43856-024-00575-y (PMC11282208; doi:10.1038/s43856-024-00575-y)
Supplement: Supplementary file 2 — Description of Additional Supplementary Files [file 43856_2024_575_MOESM2_ESM.pdf]

Description of Additional Supplementary Files

File Name: Supplementary Data 1
Description: Source data for Figure 2 and Figure 3

File Name: Supplementary Data 2
Description: Summary of serum chemistry at baseline and postdose

File Name: Supplementary Data 3
Description: Summary of hematology measurements at baseline and postdose
