# Supplementary material for: Safety and tolerability of the protein C activator AB002 in end-stage renal disease patients on hemodialysis: a randomized phase 2 trial
Source: Commun Med (Lond). 2024 Jul 26;4:153. doi: 10.1038/s43856-024-00575-y (PMC11282208; doi:10.1038/s43856-024-00575-y)
Supplement: Supplementary file 6 — Reporting Summary [file 43856_2024_575_MOESM6_ESM.pdf]

Corresponding author(s): Norah Verbout
Last updated by author(s): 01/29/2024

Reporting Summary

Nature Portfolio wishes to improve the reproducibility of the work that we publish. This form provides structure for consistency and transparency in reporting. For further information on Nature Portfolio policies, see our Editorial Policies and the Editorial Policy Checklist.

Statistics

For all statistical analyses, confirm that the following items are present in the figure legend, table legend, main text, or Methods section.

n/a	Confirmed	
☐	☒	The exact sample size (*n*) for each experimental group/condition, given as a discrete number and unit of measurement
☐	☒	A statement on whether measurements were taken from distinct samples or whether the same sample was measured repeatedly
☐	☒	The statistical test(s) used AND whether they are one- or two-sided<br>*Only common tests should be described solely by name; describe more complex techniques in the Methods section.*
☒	☐	A description of all covariates tested
☐	☒	A description of any assumptions or corrections, such as tests of normality and adjustment for multiple comparisons
☐	☒	A full description of the statistical parameters including central tendency (e.g. means) or other basic estimates (e.g. regression coefficient) AND variation (e.g. standard deviation) or associated estimates of uncertainty (e.g. confidence intervals)
☐	☒	For null hypothesis testing, the test statistic (e.g. *F*, *t*, *r*) with confidence intervals, effect sizes, degrees of freedom and *P* value noted<br>*Give P values as exact values whenever suitable.*
☒	☐	For Bayesian analysis, information on the choice of priors and Markov chain Monte Carlo settings
☒	☐	For hierarchical and complex designs, identification of the appropriate level for tests and full reporting of outcomes
☒	☐	Estimates of effect sizes (e.g. Cohen's *d*, Pearson's *r*), indicating how they were calculated

Our web collection on statistics for biologists contains articles on many of the points above.

Software and code

Policy information about availability of computer code

Data collection	Computer applications used to acquire and derive data for APC-PCI included Thermo Scientific Watson LIMS 7.6.1 HF2, Molecular Devices Softmax Pro (version 6.5.1 GxP), and Microsoft Excel. Computer applications used for Protein C, TAT, included Softmax Pro (version 5.4.2) and Microsoft Excel. Computer applications used for Potassium/Iron collection included Microsoft Excel.
Data analysis	Statistical analysis for visual clotting score, TAT, Potassium/Iron, Protein C was done using SigmaPlot 11.2 or GraphPad Prism 5. Descriptive statistics for safety data and APC-PCI was performed by Celerion using SAS version 9.3 or higher.

For manuscripts utilizing custom algorithms or software that are central to the research but not yet described in published literature, software must be made available to editors and reviewers. We strongly encourage code deposition in a community repository (e.g. GitHub). See the Nature Portfolio guidelines for submitting code & software for further information.

Data

Policy information about availability of data

All manuscripts must include a data availability statement. This statement should provide the following information, where applicable:

- Accession codes, unique identifiers, or web links for publicly available datasets
- A description of any restrictions on data availability
- For clinical datasets or third party data, please ensure that the statement adheres to our policy

Deidentified individual participant data underlying the reported results will be made available 3 months after publication for a period of 5 years. Proposals for access should be sent to: norah.verbout@aronorabio.com. The study protocol is included as a data supplement available with the online version of this article.

Research involving human participants, their data, or biological material

Policy information about studies with human participants or human data. See also policy information about sex, gender (identity/presentation), and sexual orientation and race, ethnicity and racism.

Reporting on sex and gender	Out of 36 total, 26 patients (72%) identified as male and 10 patients (28%) identified as female. Gender identity information was not collected from patients in this study.
Reporting on race, ethnicity, or other socially relevant groupings	The baseline racial groupings were as follows: 33 patients (91.6%) identified as Black or African American and 3 patients (8.3%) identified as White. The baseline ethnic groupings were as follows: 35 patients (97.2%) identified as Not Hispanic or Latino and 1 patient (2.7%) identified as Hispanic or Latino.
Population characteristics	The mean (SD) age was 54.2 (9.91) years old, the mean (SD) weight was 86.3 (21.63) kg. The average (SD) time on dialysis was 7.4 (5.90) years. The primary causes of kidney failure were hypertension (44.4%), diabetes (25%), polycystic kidney disease (3%), unknown (3%), and multiple (25%). Patient comorbidities included hypertension (94.4%), diabetes (52.8%), cardiovascular disease (33.3%), history of extremity amputation (25%), and cancer (3%).
Recruitment	Study participants were recruited from the Orlando, FL region using a site database and media advertisements.
Ethics oversight	The clinical trial protocol and all amendments were approved by IntegReview IRB institutional review board (now operating under Advarra) prior to study start and informed consent was obtained from all participants prior to study-specific procedures.

Note that full information on the approval of the study protocol must also be provided in the manuscript.

Field-specific reporting

Please select the one below that is the best fit for your research. If you are not sure, read the appropriate sections before making your selection.

☒ Life sciences ☐ Behavioural & social sciences ☐ Ecological, evolutionary & environmental sciences

For a reference copy of the document with all sections, see nature.com/documents/nr-reporting-summary-flat.pdf

Life sciences study design

All studies must disclose on these points even when the disclosure is negative.

Sample size	The sample size chosen for this study was selected without statistical considerations. The sample size of 36 patients in total (18 per cohort with a ratio of 2:1 of active study drug:matching placebo) was designed to meet the study safety, tolerability, and PD objectives.
Data exclusions	Data that were were excluded from analysis include the following conditions: 1) Dialyzers in which the plastic cartridge encasing the fibers was breached were excluded from ion concentration analysis; 2) The interquartile range method was used to assess outliers in the TAT data.
Replication	Assays were run with replicate samples.
Randomization	A statistician generated a random allocation sequence for each cohort at a 2:1 allocation ratio of drug and placebo using a computerized randomization scheme. In each cohort, patients were assigned a unique randomization number at the time of dosing, and received the corresponding treatment according to the randomization schedule, with male patients assigned consecutively upward starting at 1 (cohort 1) and 19 (cohort 2), and female patients assigned consecutively downward starting at 19 (cohort 1) and 36 (cohort 2). Block randomization was not utilized.
Blinding	Blinding of the study was maintained as follows: the randomization schedule was available only to the clinical site pharmacy staff preparing the drug who were not involved in any other aspect of the study, including drug administration. The randomization schedule was not available to the Sponsor, patients, study PI, or staff members responsible for monitoring and evaluating patients. All data were evaluated under blinded conditions. The study drug and the matching placebo were indistinguishable in appearance.

Reporting for specific materials, systems and methods

We require information from authors about some types of materials, experimental systems and methods used in many studies. Here, indicate whether each material, system or method listed is relevant to your study. If you are not sure if a list item applies to your research, read the appropriate section before selecting a response.

Materials & experimental systems

n/a	Involved in the study
☒	☐ Antibodies
☒	☐ Eukaryotic cell lines
☒	☐ Palaeontology and archaeology
☒	☐ Animals and other organisms
☐	☒ Clinical data
☒	☐ Dual use research of concern
☒	☐ Plants

Methods

n/a	Involved in the study
☒	☐ ChIP-seq
☒	☐ Flow cytometry
☒	☐ MRI-based neuroimaging

Clinical data

Policy information about clinical studies

All manuscripts should comply with the ICMJE guidelines for publication of clinical research and a completed CONSORT checklist must be included with all submissions.

Clinical trial registration

NCT03963895

Study protocol

The study protocol is included as a data supplement available with the online version of this article.

Data collection

The study was performed at the Orlando Clinical Research Center (Orlando, FL) between July 5, 2019, and December 29, 2020. Safety and efficacy data were collected at the Orlando Clinical Research Center. Additional data were collected and analyzed post-study at Aronora, Inc. (Protein C, Blood entrapment in dialyzers), HTI/Prolytix (Anti-drug antibodies), Celerion (APC-PCI) and Oregon Health & Science University (ion entrapment in dialyzers).

Outcomes

The primary outcome of this study was the safety and tolerability of AB002. Safety and tolerability outcome measures were evaluated following active treatment versus pre-treatment and placebo and included the number and severity of AEs (including vascular access site reactions), physical examinations, bleeding time from the vascular access sites, vital sign measurements (body temperature, respiratory rate, blood pressure, and heart rate), 12-lead ECGs, clinical laboratory parameters, including hematology (hemoglobin, hematocrit, total and differential leukocyte count, red blood cell count, platelet count), serum chemistry (blood urea nitrogen, chloride, bicarbonate, albumin, creatinine, glucose, alkaline phosphatase, alanine aminotransferase, aspartate aminotransferase, direct bilirubin, total bilirubin, lactate dehydrogenase, and sodium), and coagulation (fibrinogen, prothrombin time/INR, prothrombin time, thrombin time, activated partial thromboplastin time). An additional safety outcome of the study included development of antibodies to AB002 or WT thrombin after drug exposure, as measured by validated anti-drug antibody assays.

A secondary outcome of this study included pharmacodynamic evaluation of a surrogate marker of drug exposure, APC-protein C inhibitor (APC-PCI) complexes. In addition, plasma protein C levels on day of dosing were evaluated as an exploratory outcome at Aronora, Inc. by ELISA (Innovative Research, Inc.) from samples collected at 0, 4, 6, and 24 h post dose. Another secondary outcome of this study included hemodialysis efficiency, determined by comparing blood urea nitrogen (BUN) and potassium levels before and after hemodialysis after AB002 treatment versus pre-treatment and placebo. The final secondary outcome included the efficacy of antithrombotic activity, as measured by thrombus accumulation within the dialyzer circuit (evaluated by visual inspection) following active treatment with AB002.

The exploratory outcomes of this study that were not prespecified in the study protocol included the effect of AB002 on the frequency and volume of saline flushes required to maintain circuit patency during hemodialysis, the frequency of hemodialysis circuit changeouts, thrombin generation during hemodialysis, and quantification of blood entrapment within the dialyzer filter at the end of hemodialysis. These exploratory outcomes were evaluated following active treatment versus non-dosing days or placebo, as described above for thrombus accumulation in the dialyzer circuit.
